# Capture and displacement-based release of the bicarbonate anion by calix[4]pyrroles with small rigid straps†

**DOI:** 10.1039/d0sc03445b

**Published:** 2020-07-24

**Authors:** Nam Jung Heo, Ju Ho Yang, Vincent M. Lynch, Byoung Joon Ko, Jonathan L. Sessler, Sung Kuk Kim

**Affiliations:** Department of Chemistry, Research Institute of Natural Science, Gyeongsang National University Jinju 660-701 Korea sungkukkim@gnu.ac.kr; Department of Chemistry, The University of Texas at Austin 105 E. 24th Street-Stop A5300 Austin Texas 78712-1224 USA sessler@cm.utexas.edu; New Drug Development Center, Osong Medical Innovation Foundation Chungbuk Korea 28160 kobjoon@kbiohealth.kr

## Abstract

Two-phenoxy walled calix[4]pyrroles **1** and **2** strapped with small rigid linkers containing pyridine and benzene, respectively, have been synthesized. ^1^H NMR spectroscopic analyses carried out in CDCl_3_ revealed that both of receptors **1** and **2** recognize only F^−^ and HCO_3_^−^ among various test anions with high preference for HCO_3_^−^ (as the tetraethylammonium, TEA^+^ salt) relative to F^−^ (as the TBA^+^ salt). The bound HCO_3_^−^ anion was completely released out of the receptors upon the addition of F^−^ (as the tetrabutylammonium, TBA^+^ salt) as a result of significantly enhanced affinities and selectivities of the receptors for F^−^ once converted to the TEAHCO_3_ complexes. Consequently, relatively stable TEAF complexes of receptors **1** and **2** were formed *via* anion metathesis occurring within the receptor cavities. By contrast, the direct addition of TEAF to receptors **1** and **2** produces different complexation products initially, although eventually the same TEAF complexes are produced as *via* sequential TEAHCO_3_ and TBAF addition. These findings are rationalized in terms of the formation of different ion pair complexes involving interactions both inside and outside of the core receptor framework.

## Abstract 2

The inherent selectivity of anion receptors can be reversed by ion pairing occurring both inside and outside of the receptor cavity.

## Graphical abstract

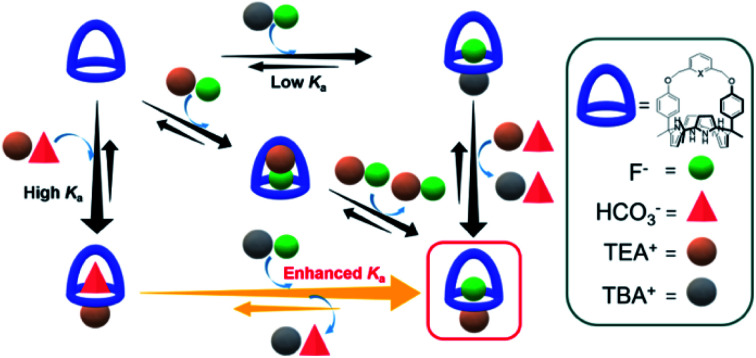

## Introduction

An increasing appreciation of the role played by anions in a variety of biological and environmental systems, as well as a number of industrial processes, has made a variety of anions specific targets for selective recognition.^1–3^ Among such anions, small, basic anions such as the bicarbonate anion (HCO_3_^−^) and the fluoride anion (F^−^) have recently drawn particular attention. For example, the bicarbonate anion plays a critical role in physiological pH buffering and signal transduction in the human central nerve system as well as in triggering certain intracellular events.^4,5^ In addition, the bicarbonate anion is an essential element in the natural carbon cycle in which the bicarbonate anion largely generated from gaseous CO_2_ from the atmosphere acts as a key component in regulating and maintaining the pH of aquatic environments; it is thus necessary for a wide range of living organisms to survive.^6^ On the other hand, the fluoride anion plays a role in public health and is key to a number of chemical processes.^7–9^ In spite of their importance, only a handful of anion receptors capable of recognizing these basic anions with near-complete selectivity have been reported.^10–13^ Moreover, to our knowledge no receptors are known whose selectivity and inherent affinity for these two specific anions, *e.g.*, the bicarbonate and fluoride anion, can be reversed by ion pairing effects. Here we report the synthesis of bicarbonate-selective receptors and the reversal of their inherent selectivity through ion pairing. As detailed below, this allows the bicarbonate anion to be bound and then released by treating with an appropriately chosen fluoride anion salt.

The design of a receptor with high selectivity for relatively basic anions, such as the bicarbonate anion or the fluoride anion, is a recognized challenge in the anion binding community.^10–12^ Good size matching between the receptor and the anion, as well as appropriate spatial preorganization and orientation of the anion binding motifs within the receptor are time-honoured approaches to achieving selectivity.^1–3,10–13^ Preorganized geometries and structural rigidity can also contribute to the preparation of receptors targeting specific anions.^1–3,10–13^ Finally, the presence of one or more auxiliary hydrogen bond donors or acceptors within the receptor framework can impart selectivity for oxoanions, such as bicarbonate, by supporting additional interactions with the anions.^13^ In aggregate, these design considerations lead us to suggest that calix[4]pyrrole, a tetrapyrrolic macrocyclic compound featuring sp^3^-hybridized *meso*-carbons, would be an attractive framework for preparing anion receptors selective for the bicarbonate anion or the fluoride anion. Calix[4]pyrroles are relatively easy to functionalize and modify synthetically.^14^ For instance, both the *meso*-carbon atoms and the β-pyrrolic carbon atoms of the calix[4]pyrrole skeleton can be substituted with various functional groups.^14^ A particularly appealing set of calix[4]pyrroles are the so-called strapped calix[4]pyrroles, wherein the two diagonally opposing *meso* carbons are linked by a tether that can be used to introduce functional groups or modulate the size and rigidity of the binding cavity.^14*c*,*g*^ For example, strapped calix[4]pyrroles bearing ancillary hydrogen bond donors on the strap show enhanced affinity for specific anions while those strapped with a small or rigid bridging element are selective for relatively small anions.^13–15^ Taking this into consideration, we designed and synthesized the HCO_3_^−^-selective anion receptors **1** and **2**. These systems consist of a calix[4]pyrrole with small rigid straps that connect two diametrical phenol walls *via* pyridine and benzene linkers, respectively. As detailed below, receptors **1** and **2** in their ion free forms are able to recognize both the bicarbonate anion (as the TEA^+^ salt) and the fluoride anion (as the TBA^+^ salt) among various test anions in CDCl_3_ with higher affinity being seen for the former anion. Counterintuitively, as compared with the ion-free forms of receptors **1** and **2**, their HCO_3_^−^ complexes were found to bind the fluoride anion (as the corresponding TBA^+^ salt) with remarkably enhanced affinity. As a result, the bound bicarbonate anion is released from receptors **1** and **2** upon the addition of the fluoride anion (as the TBA^+^ salt) *via* anion metathesis. In contrast, the direct addition of TEAF to receptors **1** and **2** produces different complexation products initially, although eventually the same TEAF complexes are produced as generated *via* sequential TEAHCO_3_ and TBAF addition. These findings are explained in terms of ion pairing effects, which are operative both inside and outside of the receptor framework.
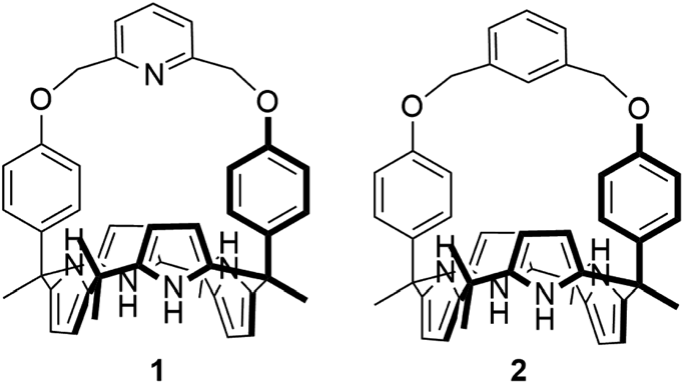


## Results and discussion

**Scheme 1:**
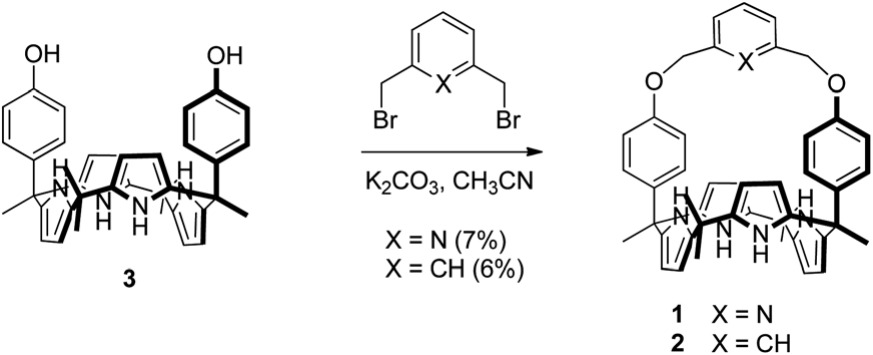
Synthesis of receptors **1** and **2**.

**Fig. 1:**
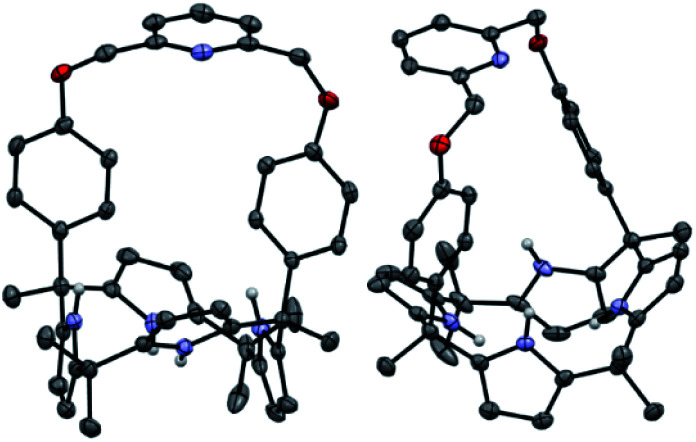
Two different views of the single X-ray crystal structure of receptor **1**. Thermal ellipsoids are scaled to the 50% probability level. Most hydrogen atoms are omitted for clarity.

Receptors **1** and **2** were prepared by following the synthetic procedure depicted in Scheme 1. In contrast to the general strategy adopted for the synthesis of a strapped calix[4]pyrrole, receptors **1** and **2** were synthesized by installing the strap units linking the diagonal *meso* carbons in the final step. Briefly, *cis*-diphenolic calix[4]pyrrole **3** was synthesized following a literature procedure *via* the [2 + 2] condensation reaction of a *meso*-phenol substituted dipyrromethane with acetone in the presence of BF_3_·OEt_2_ as a Lewis acid. It was separated from the reaction mixture, which also contains the corresponding *trans*-isomer, by column chromatography.^16^ Subsequently, compound **3** was subjected to reaction with 2,6-bis(bromomethyl)pyridine and 1,3-bis(bromomethyl)benzene in the presence of K_2_CO_3_ in acetonitrile to give the strapped calix[4]pyrroles **1** and **2**, respectively. Receptors **1** and **2** were fully characterized by standard spectroscopic means, such as ^1^H and ^13^C NMR spectroscopy and high resolution mass spectrometry (HRMS). The structure of receptor **1** was further confirmed by a single crystal X-ray diffraction analysis (Fig. 1). Single crystals of receptor **1** suitable for X-ray diffraction analysis were grown by subjecting a chloroform/methanol mixture containing receptor **1** to slow evaporation. The resulting crystal structure revealed that in the solid state the calix[4]pyrrole subunit of receptor **1** exists in the 1,3-alternate conformation with no solvent molecules found to interact with the receptor (Fig. 1).

The ability of receptors **1** and **2** to bind certain anions was assessed initially by means of ^1^H NMR spectroscopic studies carried out in CDCl_3_. When the receptors were treated with various anions (>10 equiv.) including F^−^, Cl^−^, Br^−^, I^−^, HCO_3_^−^, H_2_PO_4_^−^, HSO_4_^−^, and SO_4_^2−^, only the F^−^ and HCO_3_^−^ anions caused the proton signals of receptors **1** and **2** to undergo chemical shift changes consistent with anion binding to the respective calix[4]pyrrole subunits (Fig. S1 and S2†). We thus infer that both receptors (**1** and **2**) bind F^−^ and HCO_3_^−^ selectively over other anions (Fig. S1 and S2†). This presumed selectivity is attributed to the small cavity size and the rigidity of the straps within receptors **1** and **2**. It is important to note that in these studies all anions other than bicarbonate were used in the form of their respective tetrabutylammonium (TBA^+^) salts. The TBA^+^ salt of bicarbonate is not commercially available and our attempts to prepare and purify it proved unsuccessful, perhaps as a consequence of facile Hofmann elimination. Thus, the tetraethylammonium (TEA^+^) salt of HCO_3_^−^ was used in the present study.

In order to obtain further insights into the anion binding features of receptors **1** and **2**, detailed ^1^H NMR spectral titrations were performed with F^−^ (as its TBA^+^ salt) and HCO_3_^−^ (as its TEA^+^ salt) in CDCl_3_. For example, when receptor **1** was subjected to titration with F^−^ and HCO_3_^−^, respectively, two sets of distinguishable signals were seen for the all observable protons in the ^1^H NMR spectra before saturation was reached (at *ca.* 2.62 and *ca.* 2.06 equiv. for F^−^ and HCO_3_^−^, respectively; Fig. S3 and S4†). These peaks are attributable to the anion-free and the anion-bound forms of receptor **1**, respectively. This leads to the conclusion that the corresponding association/dissociation equilibria are slow on the ^1^H NMR time scale, as typically seen for calix[4]pyrrole with high anion affinities. The presumed strong binding interactions between receptor **1** and F^−^ and HCO_3_^−^ were further supported by the observation of anion-induced chemical shift changes in the proton signals of receptor **1**. For instance, upon the exposure of receptor **1** to F^−^, the pyrrolic NH proton signal in the ^1^H NMR spectrum initially appearing as a singlet at *δ* ≈ 6.83 ppm undergoes a remarkable downfield shift to *δ* ≈ 12.56 ppm (Δ*δ* ≈ 5.73 ppm) and becomes split into a doublet (*J* = 44.6 Hz), as would be expected for a relatively symmetric system with ^19^F–^1^H coupling (Fig. S3†). In contrast, addition of F^−^ causes the aromatic proton signals corresponding to the phenoxy walls (H_d_) and β-pyrrolic protons (H_e_) to move towards higher field (Δ*δ* ≈ 0.13 ppm for H_d_ and Δ*δ* ≈ 0.19 ppm for H_e_, respectively) (Fig. S3†). Similar ^1^H NMR spectral changes were seen when receptor **1** was titrated with HCO_3_^−^, although a small quantity of HCO_3_^−^ with respect to F^−^ was needed to achieve saturation (Fig. S4†). On the basis of these ^1^H NMR spectral titrations, the binding constants (*K*_a_) corresponding to the interaction of receptor **1** with F^−^ and HCO_3_^−^ were calculated to be approximately 240 and 1440 M^−1^, respectively (Table 1).^17^

**Table 1:** Binding constants (*K*_a_, M^−1^) corresponding to the interaction between receptors **1** and **2** and their TEAHCO_3_ complexes and the fluoride and bicarbonate anions as determined in CDCl_3_ at room temperature. The values were obtained by averaging *K*_a_ values calculated from ^1^H NMR spectral titrations with the anion salts in question

Guest	Host			
	**1**	**2**	**1**·TEAHCO_3_	**2**·TEAHCO_3_
TBAF	240	910	**3330**	**5450**
TEAF	380	980	340	430
TEAHCO_3_	**1440**	**2300**	—	—

The relatively high selectivity of receptor **1** for HCO_3_^−^ over F^−^ is attributed, in part, to the fact that the counter cation, TEA^+^, used in the case of HCO_3_^−^ possesses a relatively high charge density with respect to the TBA^+^ cation employed in the case of F^−^. For the bicarbonate complex, the TEA^+^ cation was believed to be co-bound with the HCO_3_^−^ anion and to reside in the cone-shaped electron rich calix[4]pyrrole pocket leading to formation a receptor-separated ion pair complex.^18^ This presumption was supported by the finding that the proton signals of the TEA^+^ counter cation underwent an appreciable upfield shift when receptor **1** was subject to titration with TEAHCO_3_ (Fig. S4 and *cf.* Fig. S5†).

**Fig. 2:**
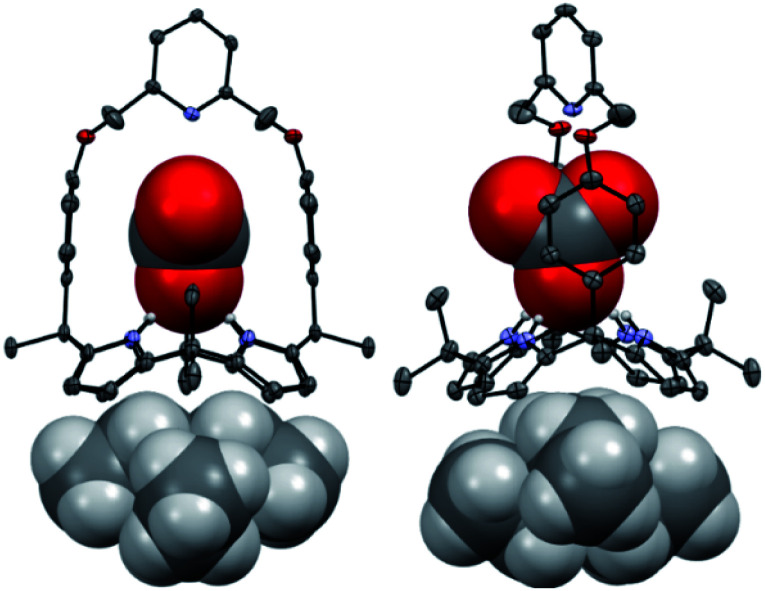
Two different views of the single X-ray crystal structure of the TEAHCO_3_ complex of receptor **1**. Thermal ellipsoids are scaled to the 30% probability level. Most hydrogen atoms are omitted for clarity.

Further evidence for the proposed binding mode of receptor **1** with TEAHCO_3_ came from a single crystal X-ray diffraction analysis (Fig. 2). Single crystals suitable for such analyses were grown by subjecting a chloroform/acetonitrile solution containing receptor **1** and an excess of TEAHCO_3_ to slow evaporation. The resulting crystal structure revealed that one oxygen atom of the HCO_3_^−^ anion forms hydrogen-bonds with all four NH protons of the calix[4]pyrrole unit at distances ranging from 2.74 and 2.95 Å (N⋯O interactions) (Fig. 2). As expected, the TEA^+^ cation is encapsulated by the electron rich calix[4]pyrrole cavity locked in the cone conformation presumably *via* electrostatic interactions (Fig. 2).

**Fig. 3:**
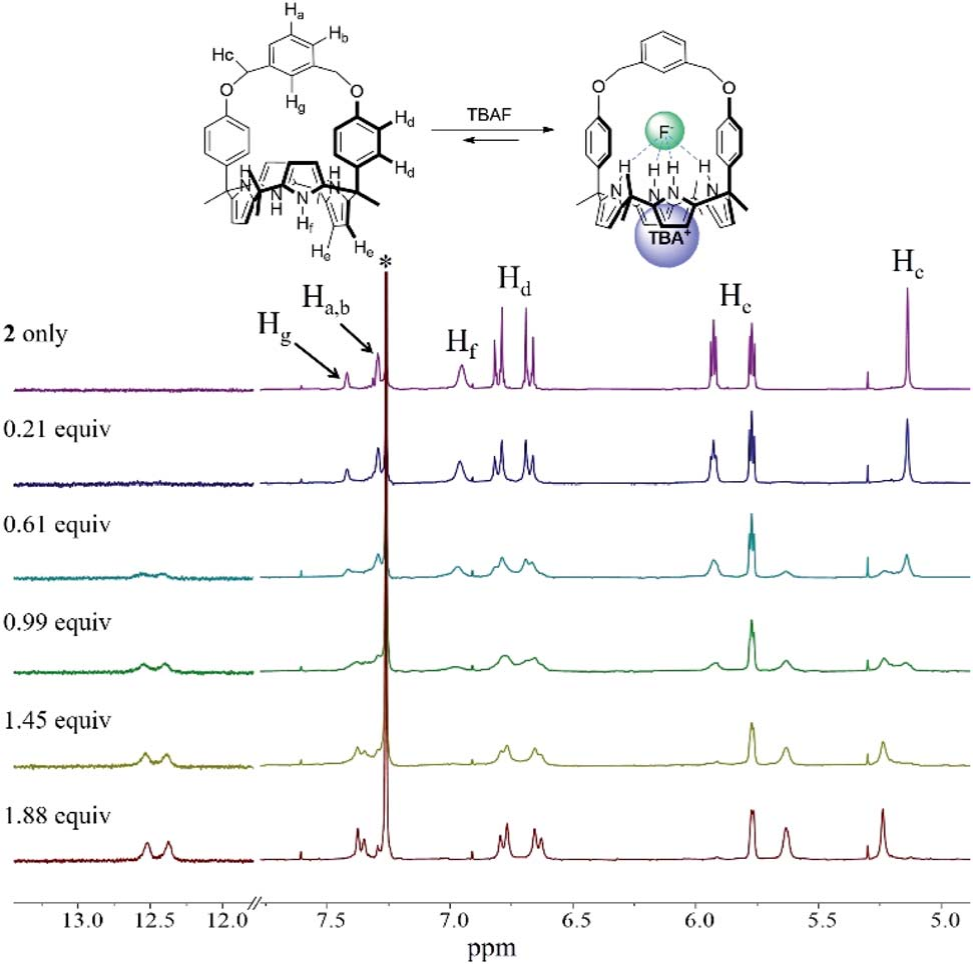
Partial ^1^H NMR spectra recorded during the titration of **2** (3 mM) with tetrabutylammonium fluoride (TBAF) in CDCl_3_. *Denotes the residual CHCl_3_ peak from the NMR solvent.

In analogy to what was seen with receptor **1**, receptor **2** (capped with benzene *in lieu* of pyridine) is able to recognize F^−^ and HCO_3_^−^*via* slow anion binding/release equilibria in CDCl_3_ (Fig. 3 and S7†). The binding constants corresponding to the interaction of receptor **2** with F^−^ and HCO_3_^−^ were estimated to be *K*_a_ = 910 M^−1^ and *K*_a_ = 2300 M^−1^, respectively (Table 1).^17^ These values were taken as an indication that receptor **2** is also selective for TEAHCO_3_ over TBAF.

The relatively high *K*_a_ values seen for HCO_3_^−^ and F^−^ in the case of receptor **2** as compared to receptor **1** are presumed to result from structural differences in the strap subunits. In the case of **1**, electron repulsion between the bound anions and the nitrogen lone pair electrons of the pyridine strap would be expected. In contrast, in the case of receptor **2** and HCO_3_^−^, an additional hydrogen bonding interaction between the anion and the aryl C–H proton (H_g_) on the strap of receptor **2** should contribute to an enhancement in the binding affinity.^19^

**Fig. 4:**
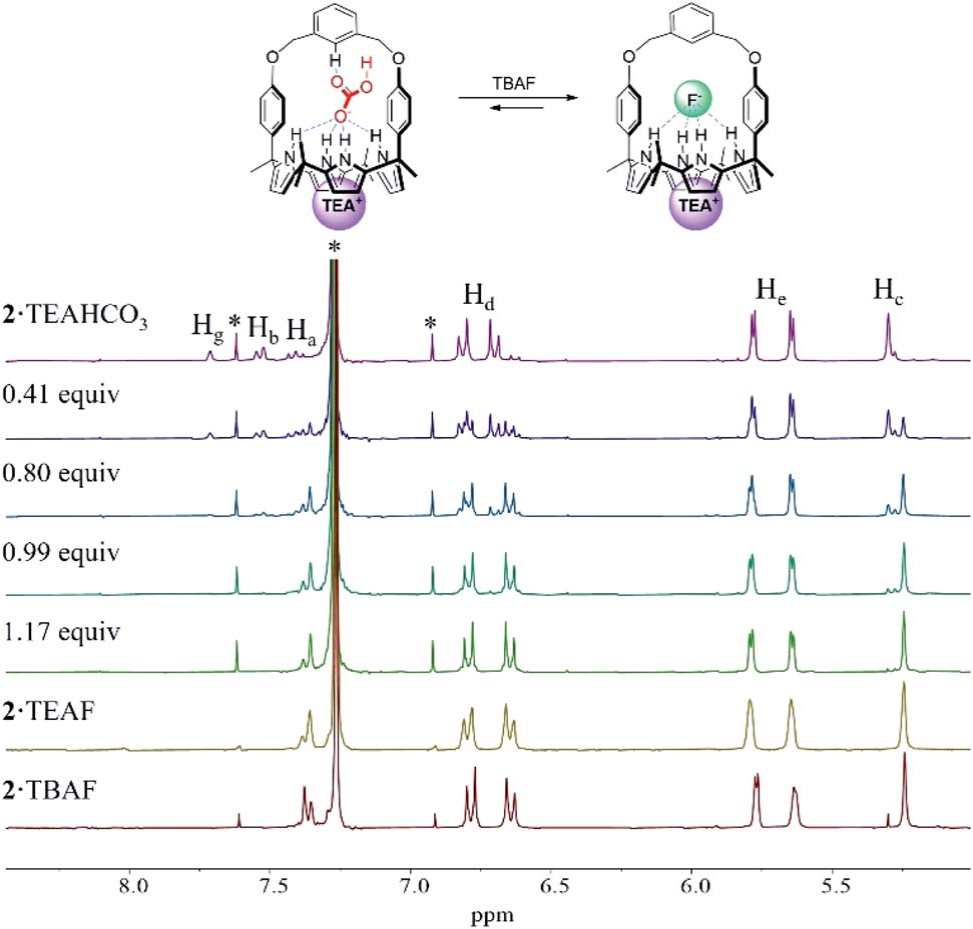
Partial ^1^H NMR spectra recorded during the titration of the TEAHCO_3_ complex of receptor **2** (**2**·TEAHCO_3_, 3 mM) with TBAF in CDCl_3_. The spectra of the TEAF and TBAF complexes of receptor **2** measured in the absence of TEAHCO_3_ are also shown. *Denotes peaks originating from the NMR solvent or NMR spinning sidebands.

In order to evaluate further the selectivity of receptors **1** and **2** for TEAHCO_3_ relative to TBAF and *vice versa*, we also quantified their affinity for F^−^ in the presence of an excess quantity of HCO_3_^−^ (as its TEA^+^ salt) by ^1^H NMR spectroscopic titrations. For instance, when the TEAHCO_3_ complex of receptor **2** (**2**·TEAHCO_3_) was titrated with F^−^ (as its TBA^+^ salt) in CDCl_3_, all proton signals corresponding to the complex of **2**·TEAHCO_3_ gradually disappeared in the ^1^H NMR spectrum giving rise, at the same time, to a new set of proton resonances consistent with the F^−^ complex of receptor **2** (Fig. 4 and S8†). These findings lead us to suggest that under these conditions the bound HCO_3_^−^ anion is released from receptor **2** and replaced by the added F^−^ anion *via* a slow exchange equilibrium to produce the corresponding **2**·TEAF complex in quantitative yield. The *K*_a_ value corresponding to the binding of F^−^ to the preformed TEAHCO_3_ complex of receptor **2** was enhanced by 6-fold and calculated to be *ca.* 5450 M^−1^ in CDCl_3_, which is remarkably high compared with what was seen when TBAF was added directly to **2** in its ion-free form (*vide supra* and Table 1).^17^ In a similar way, the HCO_3_^−^ anion complexed with receptor **1** was also readily released from the receptor cavity upon the addition of the F^−^ anion with attendant formation of the corresponding TEAF complex (**1**·TEAF). Again, this finding leads us to suggest that the preformed TEAHCO_3_ complex (**1**·TEAHCO_3_) is able to bind the F^−^ anion with a 14-fold enhanced affinity relative to the corresponding ion free form (Fig. S9† and Table 1).

The enhancement in the *K*_a_ value is rationalized in terms of F^−^ binding to receptors **1** and **2** being enhanced by strong ion pairing with the pre-bound TEA^+^ cation (*i.e.*, the counter cation added with the initial HCO_3_^−^ anion). The tetraethylammonium cation with its relatively high charge density is expected to form stronger receptor-separated ion pair complexes than its TBA^+^ congener thus enhancing the apparent fluoride anion affinity (for a discussion of the ion pairing between F^−^ and TEA^+^ and the receptors of this study, see Section S1 in the ESI†). The net result of this ion pairing is anion metathesis between the initially bound HCO_3_^−^ anion and the added F^−^ anion within the strapped cavities of these two receptors. Also contributing to an increase in the *K*_a_ value is the fact that pre-complexation of TEAHCO_3_ serves to lock the calix[4]pyrrole moiety into the cone conformation favourable for anion binding. (For a discussion of TEA^+^ cation interactions with the cavity formed when calix[4]pyrrole **2** is locked in its cone conformation, see Section S2 in the ESI†).

**Fig. 5:**
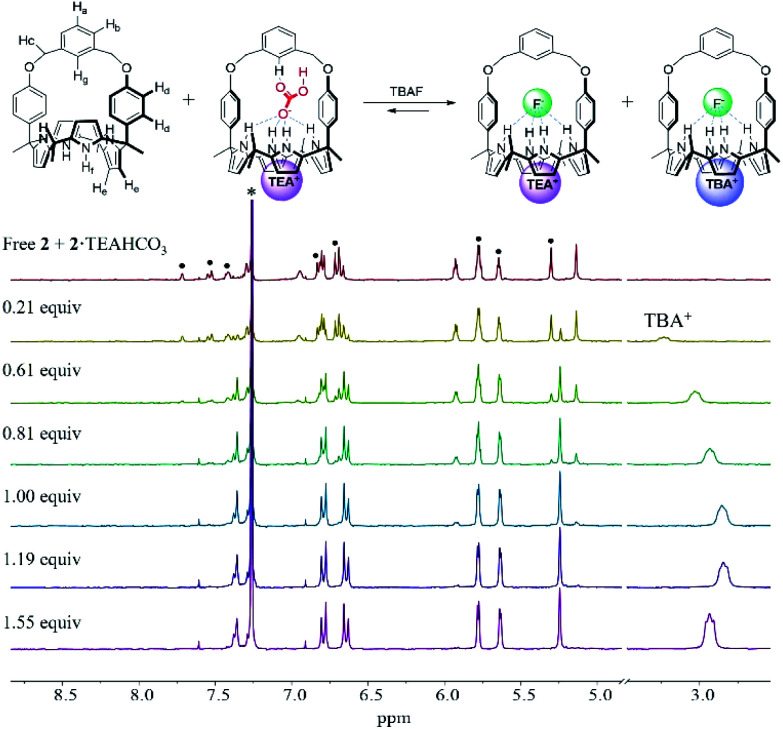
Top: Proposed fluoride complexes formed when a mixture of receptor **2** in its ion-free form and its TEAHCO_3_ complexed form are subject to titration with TBAF. Bottom: Partial ^1^H NMR spectra recorded during the titration of a mixture of ion-free **2** and its TEAHCO_3_ complex with TBAF in CDCl_3_. ·Denotes proton signals corresponding to the TEAHCO_3_ complex of receptor **2**. *Denotes peaks originating from the NMR solvent.

Further evidence that receptors **1** and **2** bind the fluoride anion *via* this proposed anion metathesis with remarkably improved efficiency came from ^1^H NMR titration experiments with the fluoride anion involving the use of 1 : 1 mixtures of the TEAHCO_3_ complexes and the anion free forms of receptors **1** and **2** (Fig. 5 and S10†). The mixtures were prepared by adding TEAHCO_3_ (0.6 equiv.) to CDCl_3_ solutions of receptors **1** and **2**, respectively. In the resulting ^1^H NMR spectra, all observable proton signals appeared as two separate sets in nearly 1 : 1 integral ratio. These peaks were assigned to the anion free and TEAHCO_3_ complexed forms of receptors **1** and **2**, respectively (Fig. 5 and S10†). When these mixtures were titrated with TBAF in CDCl_3_, a new set of proton signals corresponding to the fluoride complex of receptor **2** appeared, while those of its ion-free form and the TEAHCO_3_ complex gradually disappeared before saturation was observed upon the addition of *ca.* 1.2 equiv. of TBAF (Fig. 5). In this case, the TEAHCO_3_ complex of receptor **2** reached saturation more quickly than its ion free form (Fig. 3 and 5). Again, this finding supports our suggestions that (1) fluoride binding to receptor **2** in its cone conformation is favoured as the result of anion metathesis involving the preformed TEAHCO_3_ complexes and that (2) this complexation process is more favourable than the ostensibly similar, but chemically distinct, alternative of adding TBAF to the initial 1,3-alternate form of the ion-free receptor.

TEA^+^ binding to the cone-shaped calix[4]pyrrole cavities of receptors **1** and **2***via* cation metathesis with TBA^+^ was also quantified by ^1^H NMR spectral titrations of the TBAF complexes of receptors **1** and **2** with TEAHCO_3_. For example, when the TBAF complexes of receptors **1** and **2** were titrated with TEAHCO_3_ in CDCl_3_, the signals corresponding to the N^+^C*H*_*2*_ protons of the TBA^+^ cation gradually shift to lower field before saturation is observed upon the addition of ≈1 equiv. of TEAHCO_3_ (Fig. S11 and S12†). By contrast, the C*H*_*3*_ protons of the TEA^+^, which resonate at *ca.* 1.35 ppm in the absence of a receptor (*cf.* Fig. S5†), are seen to resonate between 0.5 and 0.7 ppm as increasing aliquots of TEAHCO_3_ are added up to ≈1 equiv. Little or no changes were observed for any of the proton signals assigned to the receptors (Fig. S11 and S12†). Collectively, these findings are consistent with the TBA^+^ cation initially located within the calix[4]pyrrole cavity being replaced by TEA^+^ as it is added in the form of the counter cation to the TEAHCO_3_ salt used in these titrations while the fluoride anion remains fully bound (*i.e.*, not displaced by the added HCO_3_^−^ anion). The *K*_a_ values corresponding to this cation metathesis were calculated to be *ca.* 23 960 M^−1^ for receptor **1** and 15 150 M^−1^ for receptor **2**, respectively.^20^ By contrast, when the ion free form of receptor **2** was treated with BF_4_^−^ (as the TEA^+^ salt), no evidence of either anion or TEA^+^ cation binding was observed (Fig. S13†). This is consistent with cation binding being dependent on anion binding and conversion of the initial 1,3-alternate conformation to the corresponding cone form.

**Fig. 6:**
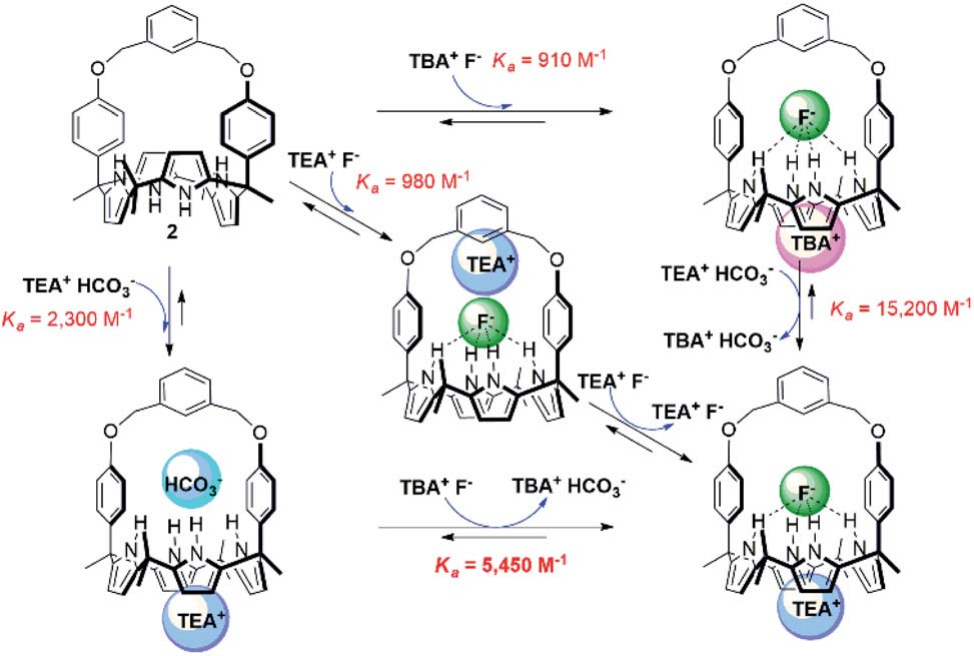
Hess diagram showing the binding modes and processes for the formation of the TEAF complex of receptor **2**.

We also investigated the effect of the counter cation on the F^−^ binding by receptors **1** and **2**, This was done by replacing the TBA^+^ cation with the TEA^+^ cation. Two sets of separate signals corresponding to the ion-free forms and TEAF complexed forms, respectively, of receptors **1** and **2** were seen in the ^1^H NMR spectra upon titration with TEAF before saturation was reached upon of the addition of 2.18 equiv. and 1.72 equiv. of TEAF, respectively (Fig. S14 and S15†). Over the course of the titrations, the binding modes of the TEAF to ion pair receptors **1** and **2** were found to change from a receptor-bound contact ion pair to receptor-separated ion pair (for a discussion of the binding of the TEAF ion pair to receptors **1** and **2**, see Section S3 in the ESI†). The binding constants (*K*_a_) of receptors **1** and **2** for the F^−^ anion as the TEA^+^ salt, estimated from the ^1^H NMR spectral titrations, were found to be 380 M^−1^ and 980 M^−1^, respectively. These values are somewhat higher than those for the corresponding TBA^+^ salt but are significantly reduced compared to those recorded when the preformed TEAHCO_3_ complexes of receptors **1** and **2** are titrated with TBAF salt (see above discussion and Table 1).^18^ This observation leads us to suggest that strong ion pairing occurs within the added TEAF salt and that this prevents in whole or in part the TEA^+^ cation from binding to the calix[4]pyrrole cavity. This reflects the greater energetics needed to separate the TEAF ion pair *vs.* forming a TEAF complex *via* addition of fluoride to a preformed TEA^+^ cation–receptor complex. As a result, TEAF initially binds to these receptors in the form of internal, as opposed to receptor-separated, ion pair complex. This sequence of binding events, which leads to the formation of the thermodynamically most stable TEAF complex of **2** in CDCl_3_, is summarised in Fig. 6.

In order to obtain greater insight into the release of bicarbonate from receptors **1** and **2** upon treatment with a fluoride anion source, we carried out ^1^H NMR spectral titrations with TEAF starting with the TEAHCO_3_ complexes of **1** and **2** (Fig. S17 and S18†). For these titrations, greater quantities of TEAF were necessary to achieve saturation than was required in the case of titration of the ion-free receptors with TEAF. This leads us to suggest that the binding affinities for F^−^ (as its TEA^+^ salt) for the TEAHCO_3_ complexes of receptors **1** and **2** are decreased with respect to those of their ion-free forms (*K*_a_ = 380 M^−1^ for ion-free **1***vs. K*_a_ = 340 M^−1^ for **1**·TEAHCO_3_ and *K*_*a*_ = 980 M^−1^ for ion-free **2***vs. K*_a_ = 430 M^−1^ for **2**·TEAHCO_3_; *cf.*Table 1). These findings stand in striking contrast to what was seen when the TEAHCO_3_ complexes of receptors **1** and **2** was titrated with F^−^ as the corresponding TBA^+^ salt (*vide supra*). Taken together, these findings provide support for the suggestions that (1) inherently strong ion pairing between TEA^+^ and F^−^ outside the receptors impedes F^−^ binding to the receptors while (2) their tendency to ion pair within the receptors favours fluoride binding. To the best of our knowledge, receptors **1** and **2** are the first examples of rationally designed receptors capable of binding ion pairs with different binding modes and affinities depending on the specific ion pair complex being formed.

**Fig. 7:**
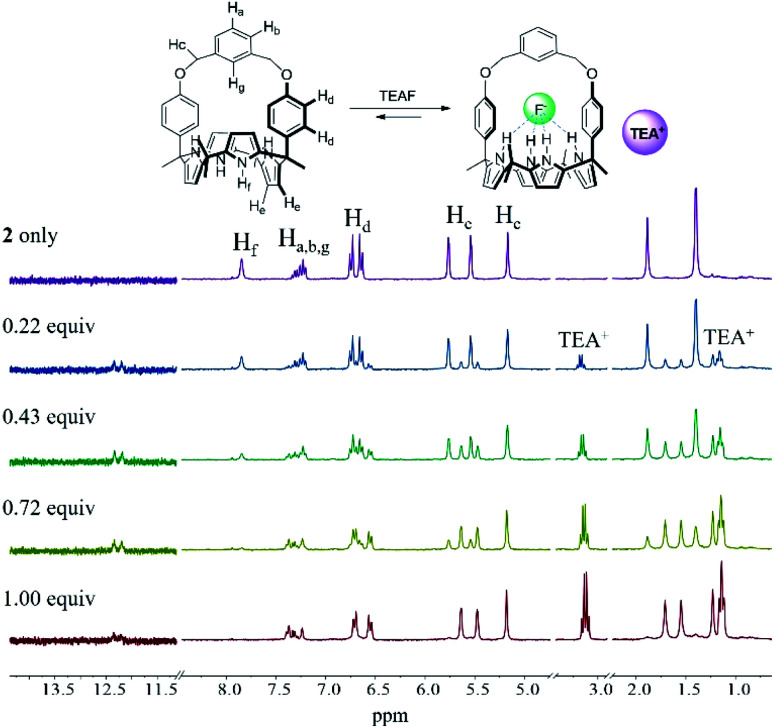
^1^H NMR spectra recorded during the titration of **2** (3 mM) with TEAF in 10% D_2_O in DMSO-*d*_6_.

In order to support the notion that tight ion pairing between TEA^+^ and F^−^ could reduce the effective fluoride anion binding affinity in the case of receptors **1** and **2**, we also performed ^1^H NMR spectral titrations involving the treatment of receptor **2** with TEAF in 10% aqueous DMSO-*d*_6_. Unfortunately, receptor **1** proved too insoluble in this medium to allow for its study. A mixture of 10% aqueous DMSO-*d*_6_ has a higher dielectric constant (*ε*_r_) than CDCl_3_ (56.2 *vs.* 4.7)^21^ and was expected to favor dissociation of TEAF into its constituent ions. On the other hand, solvation of the individual ions was expected to increase. The balance between these competing effects was not known *a priori*. Flood and coworkers reported that the anion affinity of a given receptor is likely to be diminished in proportion to the solvent dielectric constant because of the solvation of the anion.^21^ However, when receptor **2** was subjected to titration with TEAF in 10% aqueous DMSO-*d*_6_, chemical shift changes consistent with F^−^ anion binding were seen in the corresponding ^1^H NMR spectra with saturation being reached upon the addition of only ≈1 equiv. of TEAF (Fig. 7). The binding constant for this equilibrium was calculated to be *K*_a_ = 10 160 M^−1^,^17^ a value that is significantly higher than what is seen in CDCl_3_ (*K*_a_ = 380 M^−1^). On this basis we conclude that the extent to which the TEAF fluoride anion source separates into its constituent ions, TEA^+^ and F^−^, plays a crucial role in regulating the effective anion affinities of the present set of receptors. In 10% aqueous DMSO-*d*_6_ no change in the chemical shift of the TEA^+^ resonances was seen as additional quantities of TEAF were added. This observation led us to suggest that in this solvent system the TEA^+^ cation remains solvated rather than being co-bound to the cone-shaped calix[4]pyrrole cavity (Fig. 7). We thus conclude that the effects of ion pairing both within and outside of receptors **1** and **2** play a critical role in regulating the observed overall anion binding affinities.

## Conclusions

In conclusion, the strapped calix[4]pyrroles **1** and **2** wherein two diametrical *meso*-phenol walls are linked *via* a rigid benzene and pyridine spacer, respectively, were found to bind the HCO_3_^−^ anion (as the TEA^+^ salt) and the F^−^ anion (as the TBA^+^ salt) with high selectivity over various other test anions in CDCl_3_. A higher affinity for the bicarbonate anion is seen over the fluoride anion under these conditions. In contrast, upon the addition of TBAF to the preformed TEAHCO_3_ complexes of receptors **1** and **2**, the bound bicarbonate anion was released and replaced by the F^−^ anion. This anion metathesis gives rise to the corresponding TEAF complexes. As compared to what is seen when TBAF is added to receptors **1** and **2** in their ion free forms, the fluoride anion binding affinities are increased when starting with the preformed TEAHCO_3_ complex, a result ascribed to the formation of a receptor separated F^−^–TEA^+^ ion pair complex. When receptors **1** and **2** in their ion free forms are titrated with TEA^+^ and F^−^ (in the form of TEAF) TEAF complexes are formed in which the binding mode of TEA^+^ varies depending on the relative quantity of TEAF present with the same complex formed *via* anion metathesis eventually being formed. However, the observed binding affinity was unexpectedly low, a result ascribed to the TEAF salt remaining ion paired in CDCl_3_. The use of a more polar medium was found to increase the *K*_a_ values dramatically. The present findings provide support for the notion that ion pairing effects, occurring both inside and outside of the receptors, can play an important role in regulating the binding interactions between synthetic receptors and their targeted ions. In the present instance, these effects can be exploited to create systems that act as bicarbonate anion receptors from which HCO_3_^−^ can be released *via* fluoride anion-mediated anion metathesis.

## Conflicts of interest

There are no conflicts to declare.

## Supplementary Material

SC-011-D0SC03445B-s001

SC-011-D0SC03445B-s002
