# Supplementary material for: Capture and displacement-based release of the bicarbonate anion by calix[4]pyrroles with small rigid straps
Source: Chem Sci. 2020 Jul 24;11(31):8288–94. doi: 10.1039/d0sc03445b (PMC8163245; doi:10.1039/d0sc03445b)
Supplement: SC-011-D0SC03445B-s001 [file SC-011-D0SC03445B-s001.pdf]

Electronic Supplementary Material (ESI) for Chemical Science.
This journal is © The Royal Society of Chemistry 2020

Supporting Information for

Capture and displacement-based release of the bicarbonate anion by calix[4]pyrroles with small rigid straps

Nam Jung Heo,[a] Ju Ho Yang,[a] Vincent M. Lynch,[b] Byoung Joon Ko,[*,c] Jonathan L. Sessler,[*,b] and Sung Kuk Kim[*,a]

[a]*Department of Chemistry and Research Institute of Natural Science, Gyeongsang National University, Jinju, 660-701, Korea,* [b]*Department of Chemistry, The University of Texas at Austin, 105 E. 24th Street-Stop A5300, Austin, Texas 78712-1224, USA, and* [c]*New Drug Development Center, Osong Medical Innovation Foundation, Chungbuk, Korea, 28160,*

General experimental and synthetic details S2 – S3

Determination of association constants S4

^{1}H NMR spectral data for the binding interactions of receptors **1** and **2** with anions and ion pairs S5 – S25

Section S1 Discussion of the ion pairing between F^- and TEA^+ within receptors **1** and **2** S11

Section S2 Discussion of the interactions between the TEA^+ cation and the cavity formed when calix[4]pyrrole **2** is locked in its cone conformation S14

Section S3 Discussion of the interactions between the TEAF ion pair with receptors **1** and **2** S23

X-ray experimental for receptor **1** S26 – S31

^{1}H and ^{13}C NMR spectra and HRMS data for structural characterization S22 – S38

References S39

General experimental and synthetic details

Solvents and reagents used for the synthetic work were purchased from Aldrich, TCI, or Alfa Aesar and used without further purification. Compound **3** was prepared as reported previously.[1] NMR spectra were recorded on a Bruker Advance-300 MHz instrument. The NMR spectra were referenced to residual solvent peaks and the spectroscopic solvents were purchased from either Cambridge Isotope Laboratories or Aldrich. Chemical ionization (CI) and electrospray ionization (ESI) mass spectra were recorded on a VG ZAB-2E instrument and a VG AutoSpec apparatus, respectively. TLC analyses were carried out using Sorbent Technologies silica gel (200 mm) sheets. Column chromatography was performed on Sorbent Technologies silica gel 60 (40–63 mm).

Receptor 1: Compound **3** (1.67 g, 2.86 mmol), 2,6-bis(bromomethyl)pyridine (0.76g, 2.86 mmol) and K_2CO_3 (1.97 g, 14.25 mmol) were dissolved in 100 mL of acetonitrile and heated to reflux under a nitrogen atmosphere. After the resulting reaction mixture was stirred at reflux for 24 hours, the volatiles were removed *in vacuo*. To the resulting brown solid, CH_2Cl_2 (75 mL) was added and the organic layer was separated off and washed three times with 100 mL of water. The organic layer was dried over anhydrous $MgSO_4$ and the solvent was evaporated *in vacuo* to give a brown solid. Column chromatography over silica gel (eluent: ethyl acetate/hexanes (1:6)) gave 0.15 g (7.6% yield) of **1** as a white solid. ^{1}H NMR (300 MHz, $CDCl_3$) δ 7.71 – 7.66 (t, *J* = 7.7 Hz, 1H, pyridine Ar*H*), 7.41 – 7.39 (d, *J* = 7.7 Hz, 2H, pyridine Ar*H*), 6.96 – 6.93 (d, *J* = 8.8 Hz, 4H, Ar*H*), 6.82 – 6.79 (d, *J* = 8.8 Hz, 4H, Ar*H*) 6.79 (broad s, 4H, pyrrolic NH), 5.97 – 5.95 (t, *J* = 3.4, 2.7 Hz, 4H, β-pyrrolic C*H*), 5.81 – 5.79 (t, *J* = 3.4, 2.7 Hz, 4H, β-pyrrolic C*H*), 5.27 (s, 4H, ArCH_2), 1.92 (s, 6H, CH_3), 1.47 (s, 6H, CH_3), 1.38 (s, 6H, CH_3). ^{13}C NMR (75 MHz, $CDCl_3$) δ 157.4, 156.8, 138.8, 138.2, 136.7, 136.6, 128.1, 122.6,

115.4, 105.0, 104.9, 72.6, 44.1, 35.6, 30.0, 29.9, 29.5. HRMS (ESI) *m/z* 688.3652 $[M+H]^+$ calcd for $C_{45}H_{46}N_5O_2$, found 688.3691.

Receptor 2: Under a nitrogen atmosphere, 2,6-bis(bromomethyl)benzene, compound **3** (0.84g, 3.18 mmol) and K_2CO_3 (2.20 g, 15.92 mmol) in 100 mL of acetonitrile were heated to reflux. After the reaction mixture was held at reflux for 24 hours, the acetonitrile was removed in vacuo. To the resulting brown solid, CH_2Cl_2 (75 mL) were added and the organic layer was separated off and washed three times with 100 mL of water. The organic layer was dried over anhydrous $MgSO_4$ and the solvent was evaporated in vacuo to give a brown solid. Column chromatography over silica gel (eluent: ethyl acetate/hexanes (1:10)) of 0.12 g (5.6 % yield) of **1** as a yellowish solid. ^{1}H NMR (300 MHz, $CDCl_3$) δ 7.42 (s, 1H, strap Ar*H*), 7.29 (m, 3H, strap Ar*H*), 6.99 (s, 4H, pyrrolic N*H*), 6.82 – 6.79 (d, J = 8.8 Hz, 4H, Ar*H*), 6.69 – 6.66 (d, J = 8.7 Hz, 4H, Ar*H*), 5.94 – 5.92 (t, J = 3.1 Hz, 4H, β-pyrrolic C*H*), 5.79 – 5.77 (t, J = 3.0 Hz, 4H, β-pyrrolic C*H*), 5.14 (s, 4H, ArCH_2), 1.94 (s, 6H, CH_3), 1.47 (s, 6H, CH_3), 1.41 (s, 6H, CH_3). ^{13}C NMR (75 MHz, $CDCl_3$) δ 157.0, 139.6, 138.8, 136.9, 129.5, 128.0, 127.3, 116.6, 105.4, 104.5, 71.7, 44.1, 35.5, 29.4, 29.3, 28.8. HRMS (ESI) *m/z* 687.3699 $[M+H]^+$ calcd for $C_{46}H_{47}N_4O_2$, found 687.3731.

Determination of association constants using ^{1}H NMR spectral titrations for a host-guest association/dissociation equilibrium that is slow on the NMR time scale

Equilibrium: $A + B \xrightleftharpoons{K_a} AB$

Equilibrium constant: $$K_a = \frac{[AB]}{[A][B]} = \frac{[AB]}{(c(A)-[AB])(c(B)-[AB])} \quad (1)$$

c(A) and c(B) are the initial concentrations of A and B, and [A], [B] and [AB] are the equilibrium concentrations of the three species.

A and B is in slow exchange with the complex AB on the ^{1}H NMR time scale.

Two signals for one specific proton on A can be seen in the spectrum, corresponding to complexed and uncomplexed forms of A:

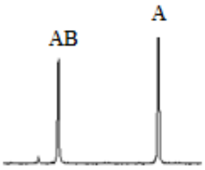

Single-point Methods

K_a is determined from the integrals of complexed and uncomplexed A. If I(A) denotes the integral of a signal for one specific proton of A and I(AB) the integral for the same proton in the complex, the concentration of AB at equilibrium is shown by eq 2. The equilibrium expression is obtrained after substituting into eq. (1):

$$[AB] = \frac{I(AB)}{I(A)+I(AB)} c(A) \quad (2)$$

$$K_a = \frac{I(AB)}{I(A)(c(B) - \frac{I(AB)}{I(A)+I(AB)} c(A))} \quad (3)$$

Binding studies of receptors 1 and 2 using ^{1}H NMR spectroscopy

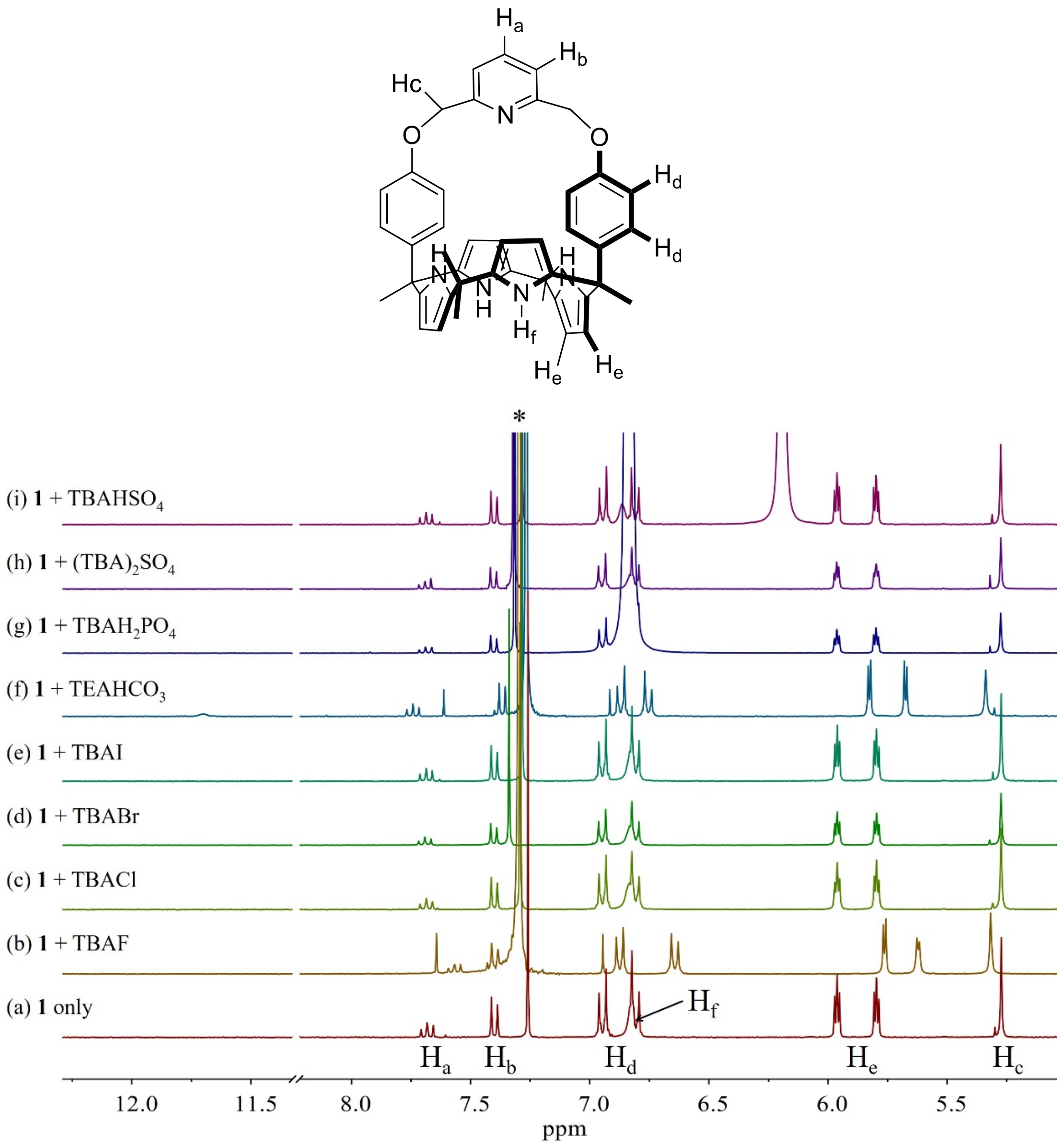

Figure S1. Partial ^{1}H NMR spectra of (a) **1** (3 mM) only, (b) **1** + excess TBAF (tetrabutylammonium fluoride), (c) **1** + excess TBACl (tetrabutylammonium chloride), (d) **1** + excess TBABr (tetrabutylammonium bromide), (e) **1** + excess TBAI (tetrabutylammonium iodide), (f) **1** + excess $TEAHCO_3$ (tetraethylammonium bicarbonate), (g) **1** + excess $TBAH_2PO_4$ (tetrabutylammonium dihydrogen phosphate), (h) **1** + excess $(TBA)_2SO_4$ (bis(tetrabutylammonium) sulfate), and (i) **1** + excess $TBAHSO_4$ (tetrabutylammonium hydrogen sulfate) in $CDCl_3$.

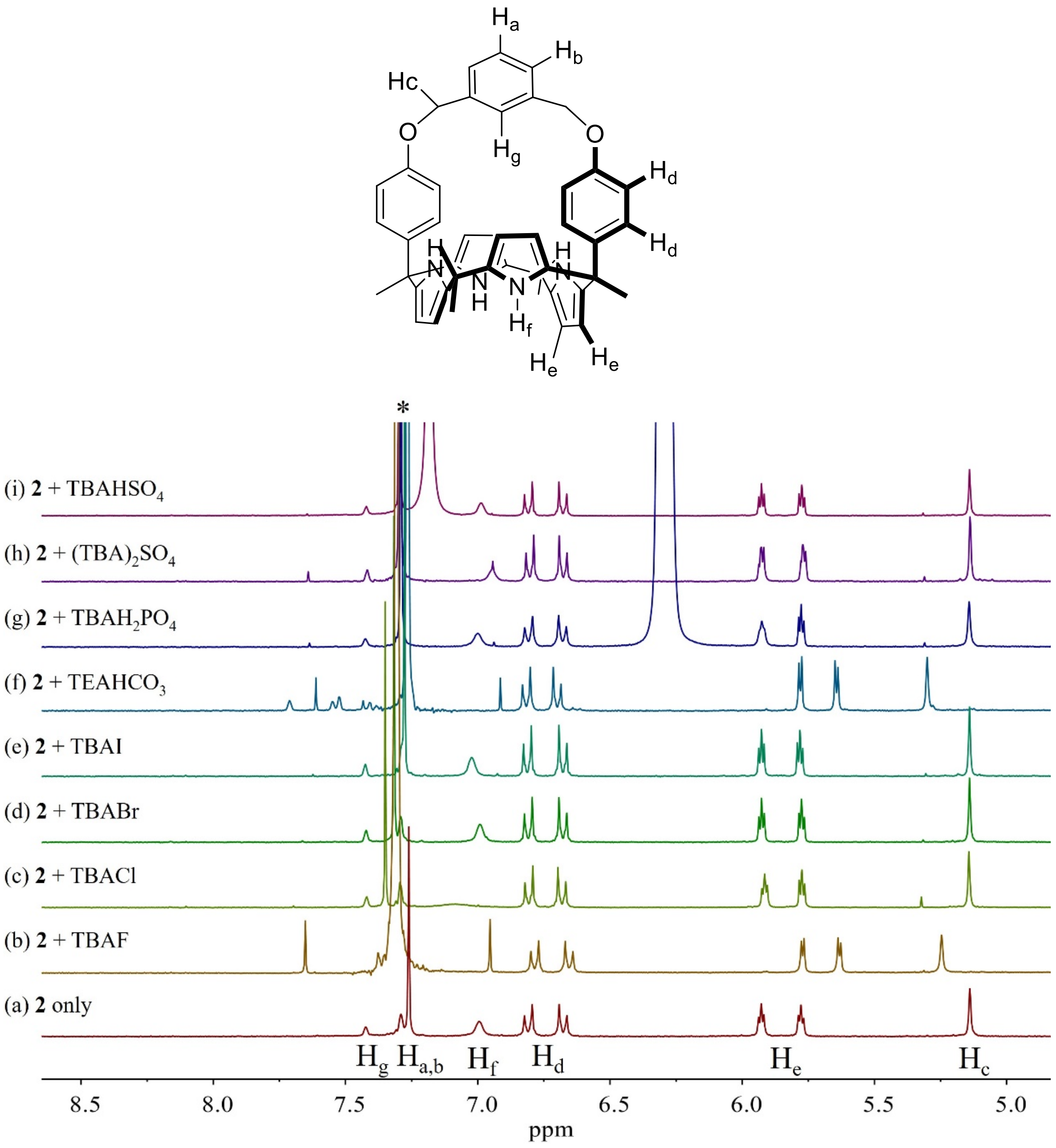

Figure S2. Partial ^{1}H NMR spectra of (a) **2** (3 mM) only, (b) **2** + excess TBAF (tetrabutylammonium fluoride), (c) **2** + excess TBACl (tetrabutylammonium chloride), (d) **2** + excess TBABr (tetrabutylammonium bromide), (e) **2** + excess TBAI (tetrabutylammonium iodide), (f) **2** + excess $TEAHCO_3$ (tetraethylammonium bicarbonate), (g) **2** + excess $TBAH_2PO_4$ (tetrabutylammonium dihydrogen phosphate), (h) **2** + excess $(TBA)_2SO_4$ (bis(tetrabutylammonium) sulfate), and (i) **2** + excess $TBAHSO_4$ (tetrabutylammonium hydrogen sulfate) in $CDCl_3$.

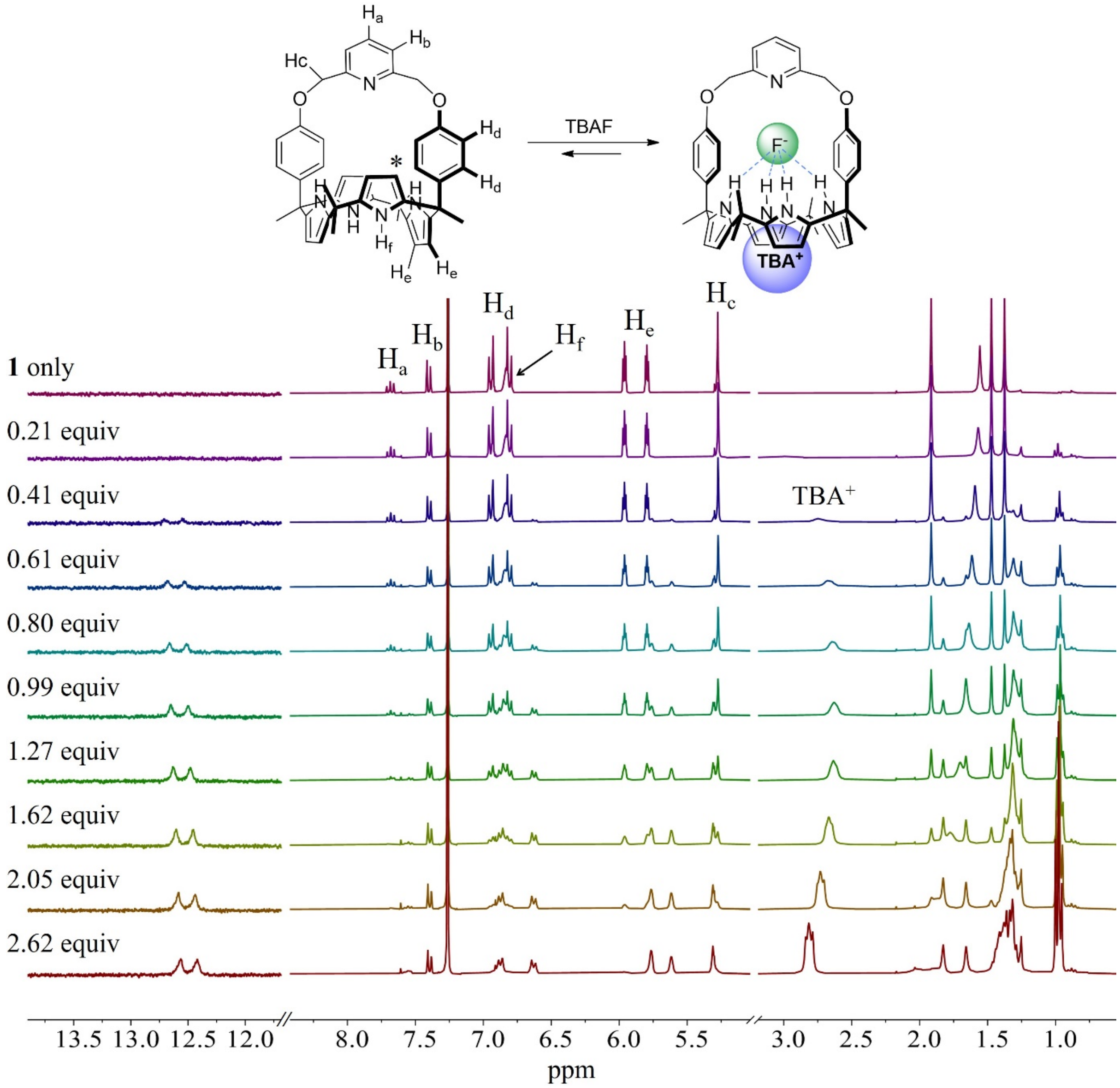

Figure S3. ^{1}H NMR spectra recorded during the titration of **1** (3 mM) with tetrabutylammonium fluoride (TBAF) in $CDCl_3$. *Denotes the residual $CHCl_3$ peak from the NMR solvent.

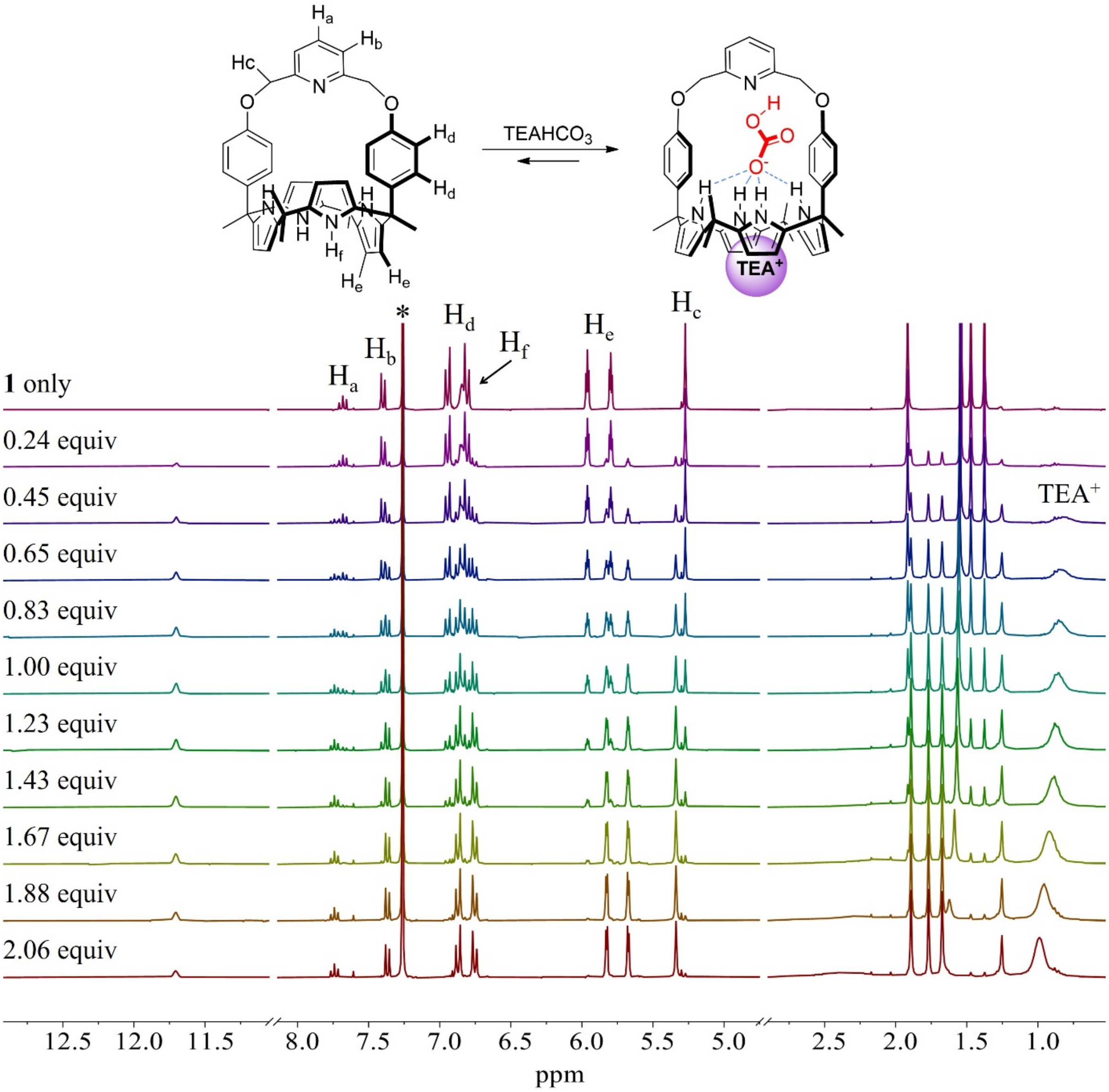

Figure S4. ^{1}H NMR spectra recorded during the titration of **1** (3 mM) with tetraethylammonium bicarbonate ($TEAHCO_3$) in $CDCl_3$. *Denotes the residual $CHCl_3$ peak from the NMR solvent.

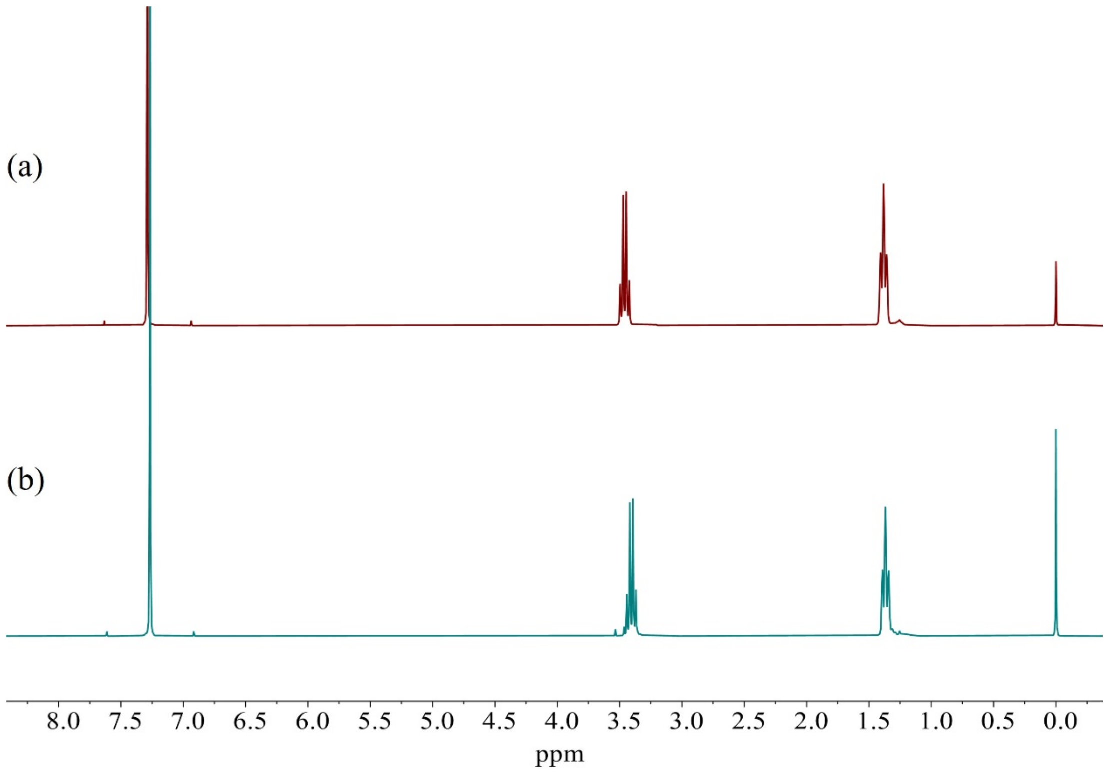

Figure S5. ^{1}H NMR spectra of (a) TEAF (tetraethylammonium fluoride) and (b) $TEAHCO_3$ (tetraethylammonium bicarbonate) recorded in the absence of the receptors in $CDCl_3$.

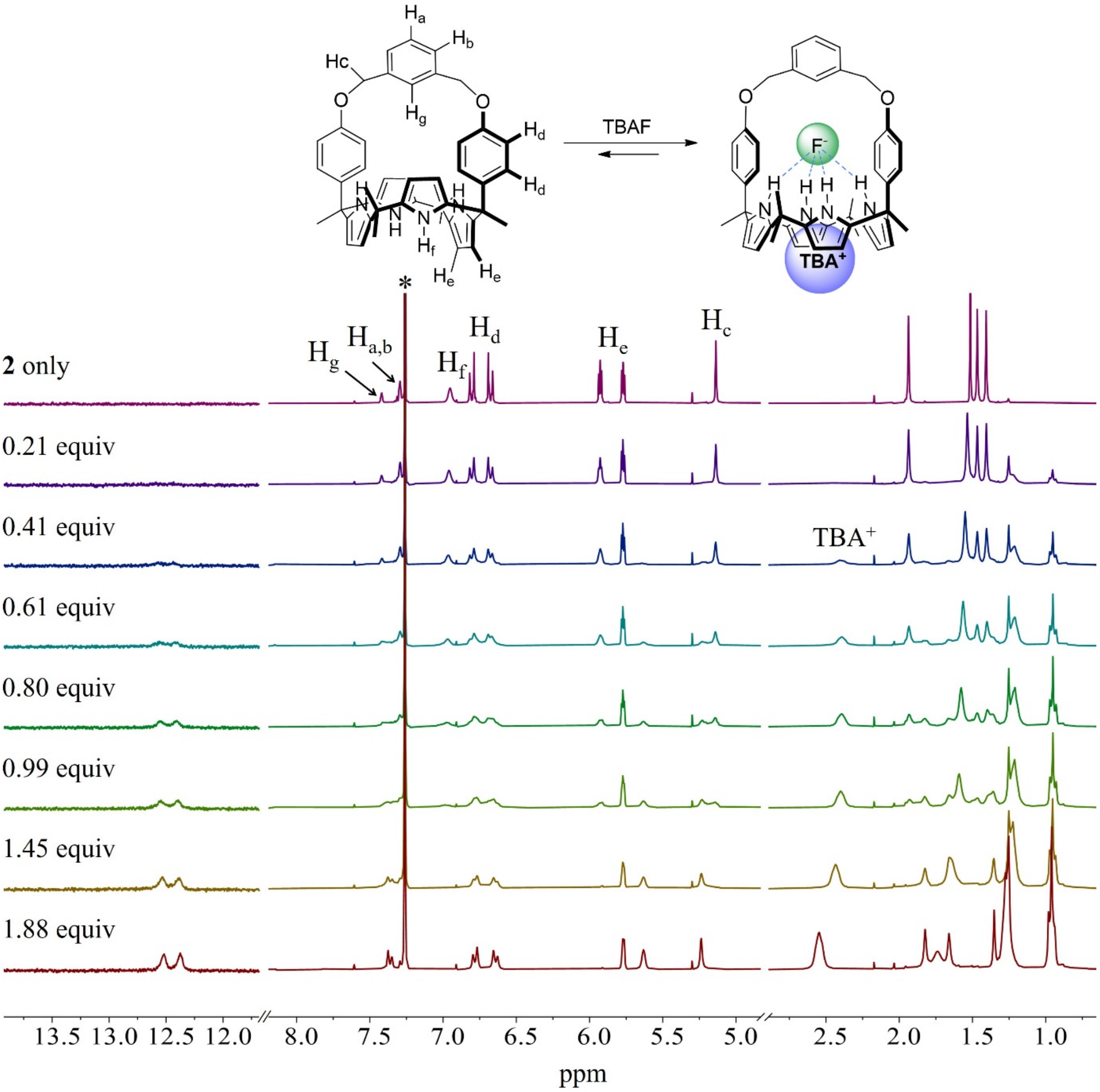

Figure S6. ^{1}H NMR spectra recorded during the titration of **2** (3 mM) with tetrabutylammonium fluoride (TBAF) in $CDCl_3$. *Denotes the residual $CHCl_3$ peak from the NMR solvent.

Section S1: Discussion of the ion pairing between F^- and TEA^+ within receptors 1 and 2

The presumption that the ion pairing between F^- and the tetraalkylammonium cations within the receptor serves to promote the binding of F^- to receptors **1** and **2** was further supported by the observation of chemical shift changes in the proton signals ascribed to the TBA^+ cation when receptors **1** and **2** were subject to a ^{1}H NMR spectral titration with TBAF in $CDCl_3$. Specifically, the proton signals corresponding to the N^+CH_2 protons of the TBA^+ counter cation appeared considerably upfield-shifted ($\delta \approx 2.67$ ppm for **1** and $\delta \approx 2.40$ ppm for **2**) before saturation was achieved (Figures S3 and S6). This finding is attributed to the TBA^+ cation being predominantly positioned within the bowl-shaped calix[4]pyrrole cavity. Over the course of the titration with TBAF, the proton signals in question gradually shift to lower field (Figures S3 and S6). Upon the titration of receptors **1** and **2** with TBAF in the presence of excess $TEAHCO_3$, the TBA^+ N^+CH_2 proton signals were seen at relatively low field ($\delta \approx 3.32$ and $\delta \approx 3.35$ ppm for **1** and **2**, respectively) with their position remaining unchanged throughout the course of the titration (Figures S8 and S9). This finding was rationalized in terms of the TBA^+ remaining outside the calix[4]pyrrole cavity because the smaller and presumably more tightly bound TEA^+ cation occupies the electron rich cup-like pyrrolic cavity with a strong ion pairing with the co-bound F^- anion.

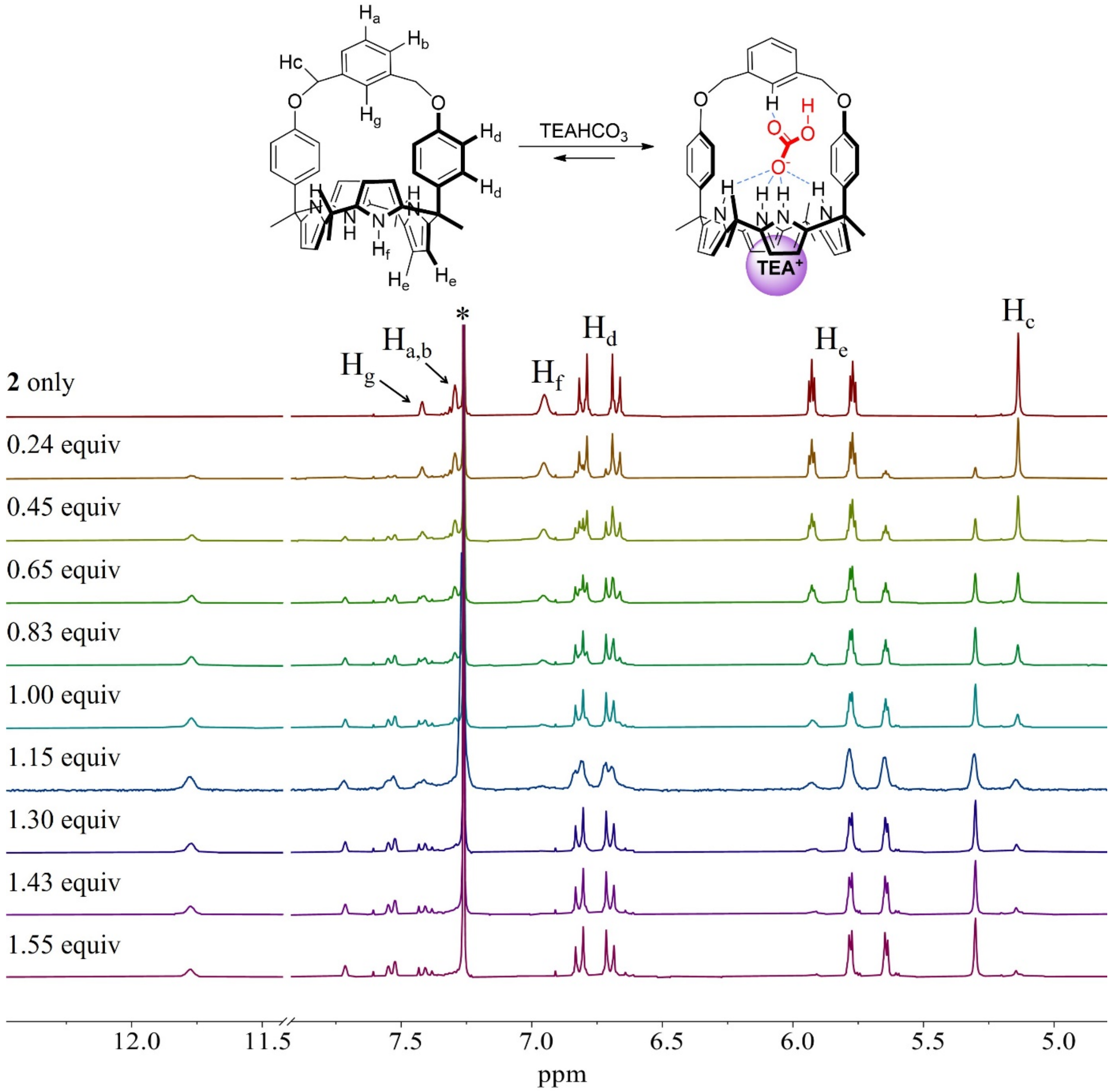

Figure S7. Partial ^{1}H NMR spectra recorded during the titration of **2** (3 mM) with tetraethylammonium bicarbonate (TEAHCO$_3$) in CDCl$_3$. *Denotes the residual CHCl$_3$ peak from the NMR solvent.

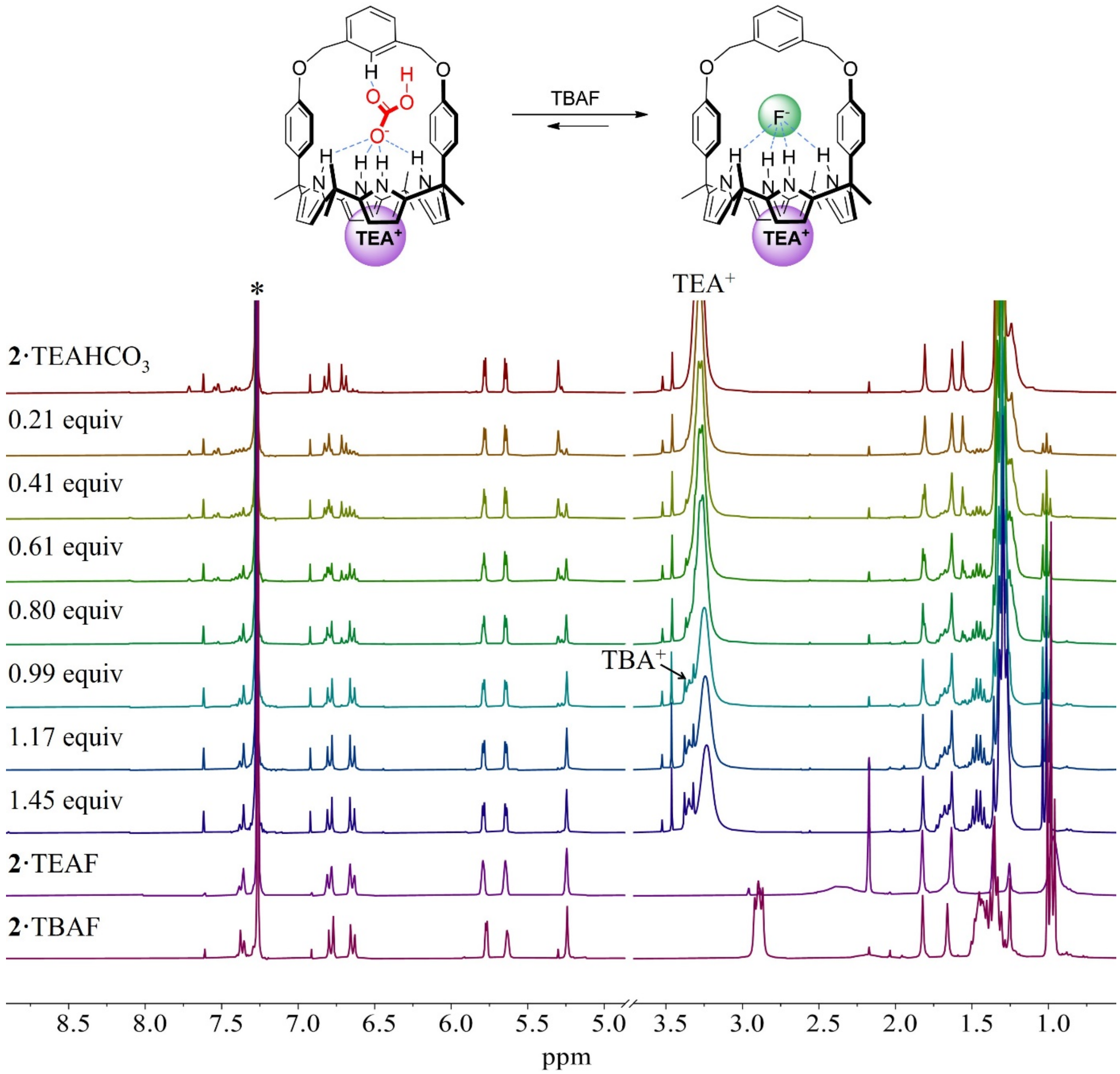

Figure S8. ^{1}H NMR spectra recorded during the titration of the $TEAHCO_3$ complex of receptor **2** (**2**·$TEAHCO_3$, 3 mM) with TBAF in $CDCl_3$. For comparison the spectra of the TEAF and TBAF complexes of receptor **2** measured in the absence of $TEAHCO_3$ are also shown. *Denotes peaks originating from the NMR solvent or impurities contained in the TBAF hydrate used for this ^{1}H NMR spectral titration.

Section S2: Discussion of the interactions between the TEA^+ cation and the cavity formed when calix[4]pyrrole 2 is locked in its cone conformation

Evidence for the TEA^+ cation pre-occupying the cone-shaped calix[4]pyrrole cavity came from the observation of chemical shift changes in the N^+CH_2 proton signals of the TBA^+ cation when a mixture of receptor **2** in its anion-free form and its $TEAHCO_3$ complex was subjected to ^1H NMR spectral titration with TBAF. As TBAF is added, the protons of the TBA^+ are seen to resonate at increasingly lower field, ultimately appearing further downfield than when the ion-free form of receptor **2** is titrated with TBAF but upfield relative to those seen when the fully preformed $TEAHCO_3$ complex is titrated with TBAF (Figures 5, S7, and S8). These observations are rationalized in terms of the co-existence of species wherein the TBA^+ either occupies the cone-shaped calix[4]pyrrole cavity of receptor **2** or remains outside the receptor cavity because it contains a pre-complexed TEA^+ cation. The net result is the formation of a mixture of **2**·TEAF and **2**·TBAF as shown in Fig. 6.

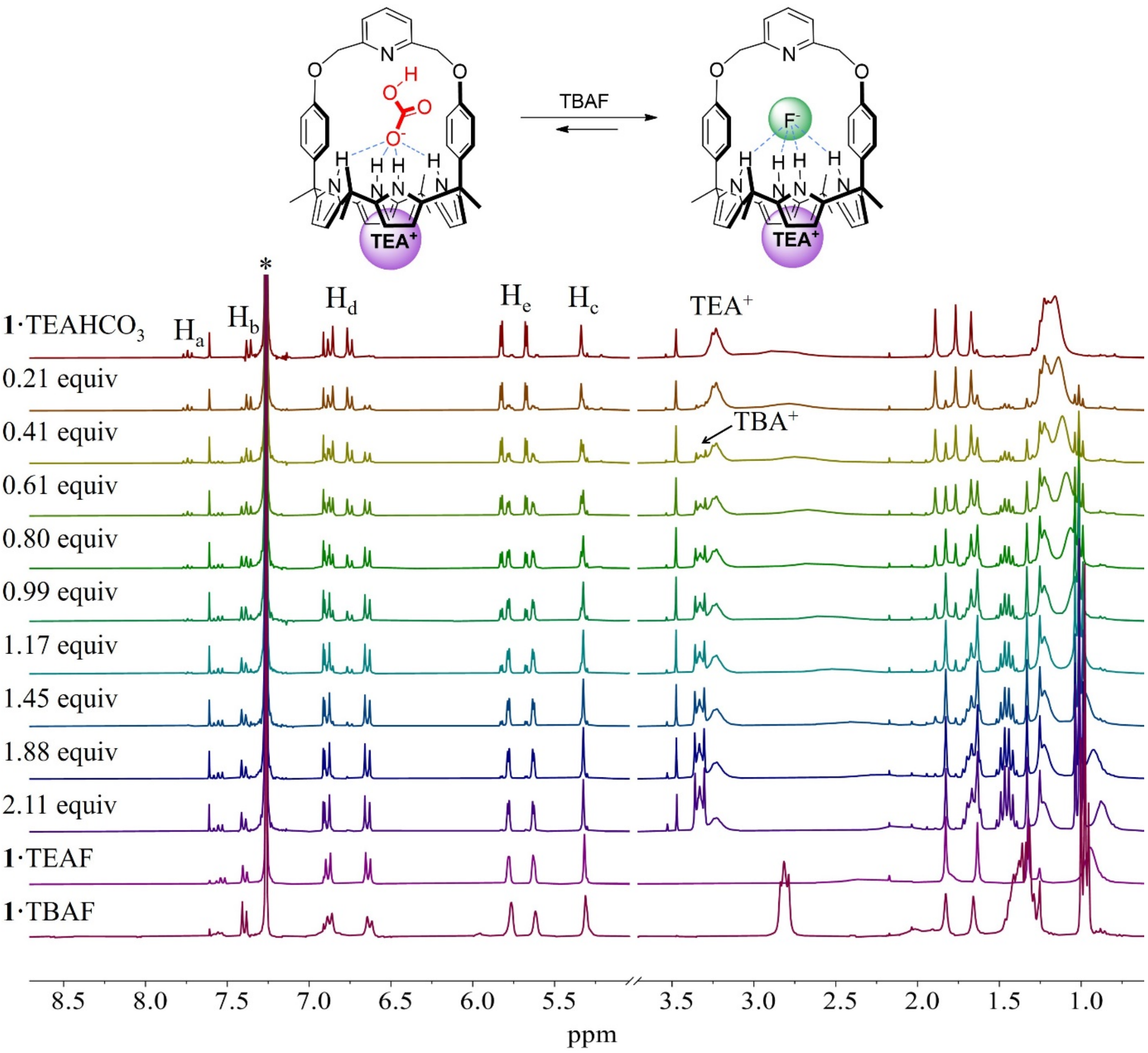

Figure S9. ^{1}H NMR spectra recorded during the titration of the $TEAHCO_3$ complex of receptor **1** (**1**·$TEAHCO_3$, 3 mM) with TBAF in $CDCl_3$. For comparison the spectra of the TEAF and TBAF complexes of receptor **1** measured in the absence of $TEAHCO_3$ are also shown. *Denotes peaks originating from the NMR solvent or impurity contained in the TBAF hydrate used for this ^{1}H NMR spectral titration.

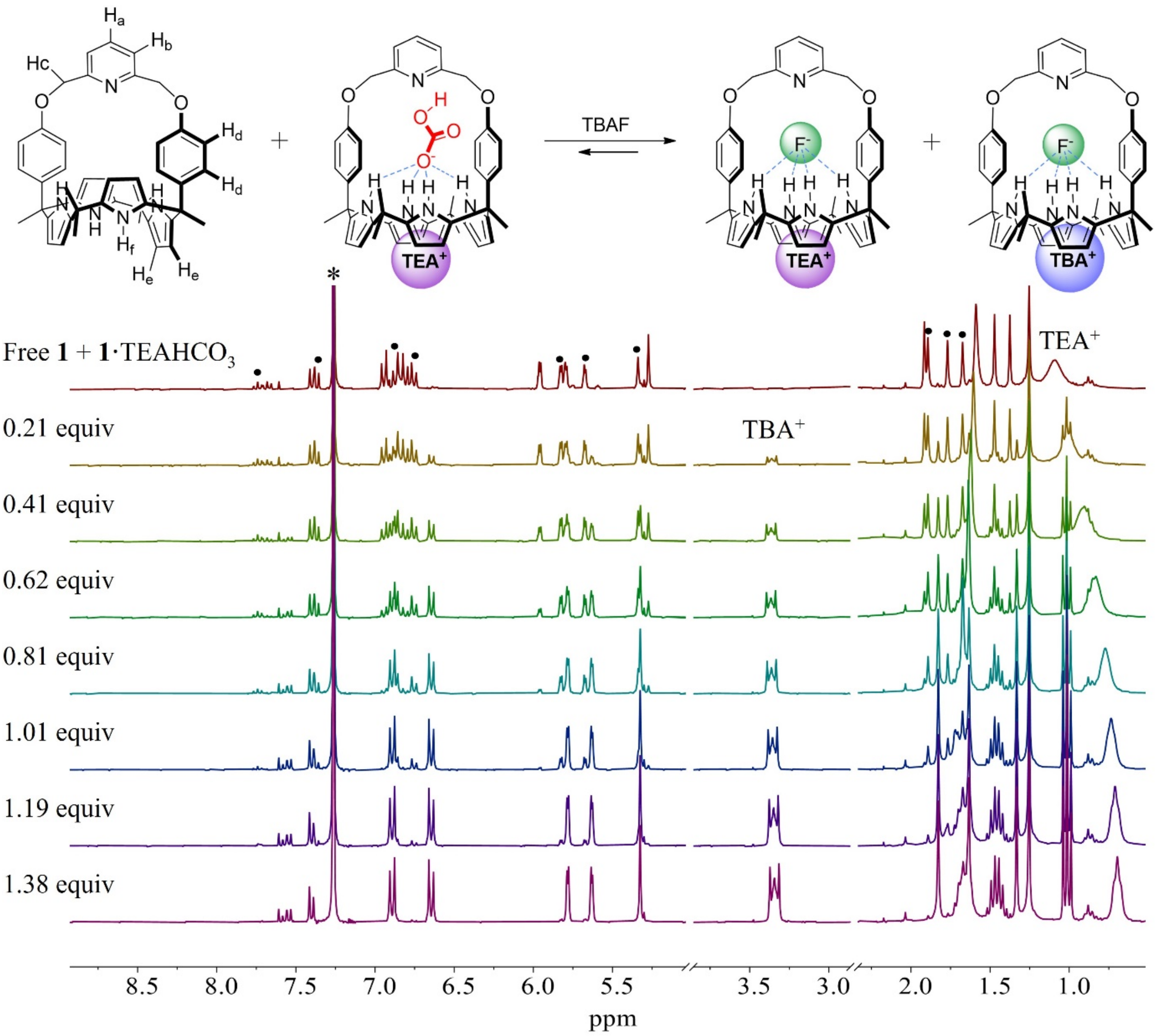

Figure S10. Top: Proposed fluoride bound products produced from a mixture of receptor **1** in its ion-free form and its $TEAHCO_3$ complex form. Bottom: Partial ^1H NMR spectra recorded during the titration of a mixture of ion-free **1** and its $TEAHCO_3$ complex with TBAF in $CDCl_3$. •Denotes proton signals corresponding to the $TEAHCO_3$ complex of receptor **1**. *Denotes peaks originating from the NMR solvent.

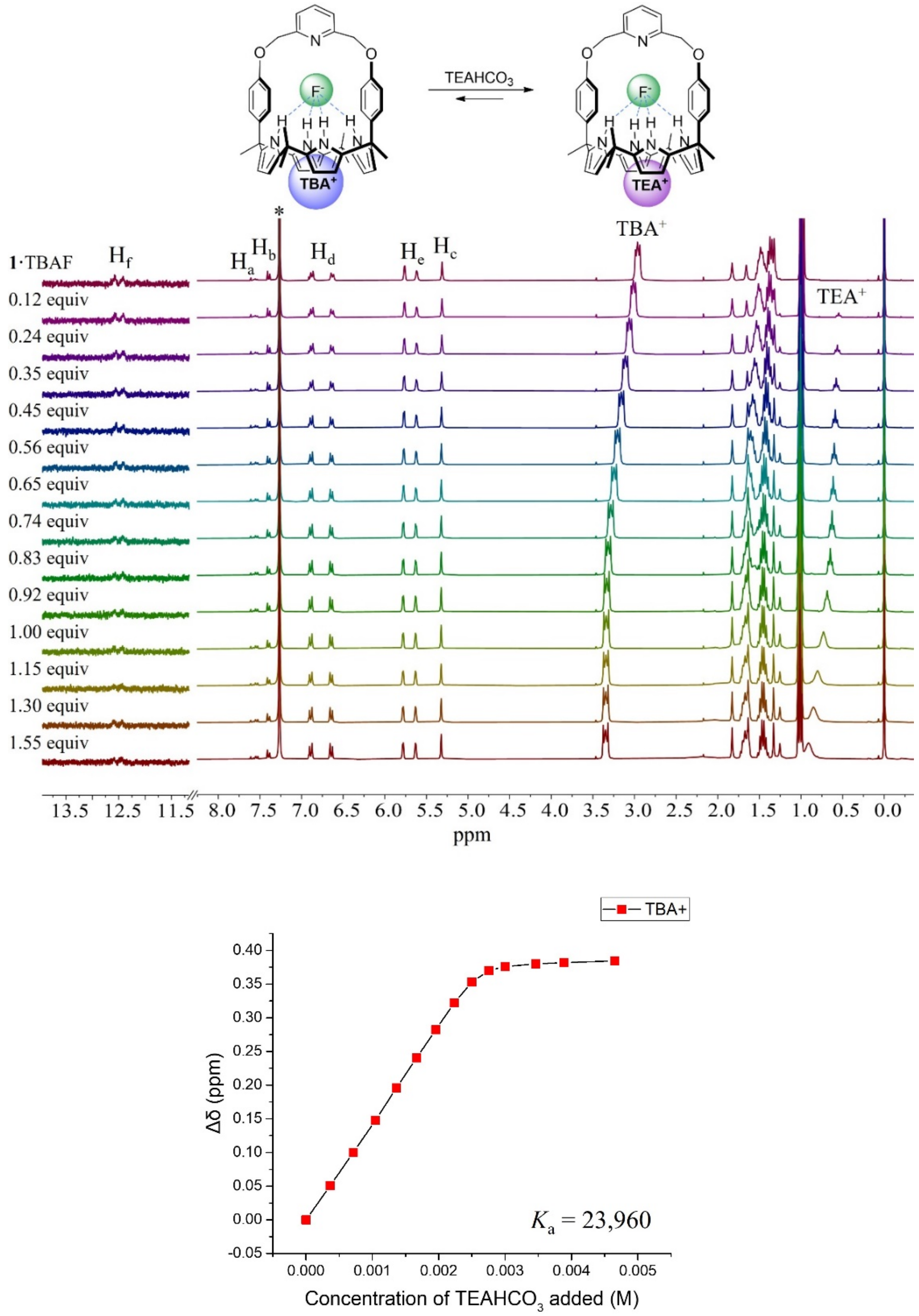

Figure S11. Top: Proposed cation exchange process between TBA^+ an TEA^+. For proton labels see Figure S9. Middle: ^1H NMR spectra recorded during the titration of the TBAF complex of receptor **1** (**1**·TBAF, 3 mM) with $TEAHCO_3$ in $CDCl_3$. Bottom: The corresponding binding isotherm for cation metathesis between TBA^+ and TEA^+. The complex of **1**·TBAF was prepared by adding 3 equiv. of TBAF to the $CDCl_3$ solution of receptor **1**. *Denotes the residual $CHCl_3$ peak from the NMR solvent. The association constant corresponding to the interaction of the TBAF complex of receptor **1** with TEA^+ was obtained using BindFit v5.0 available from URL: "http://app.supramolecular.org/bindfit/".

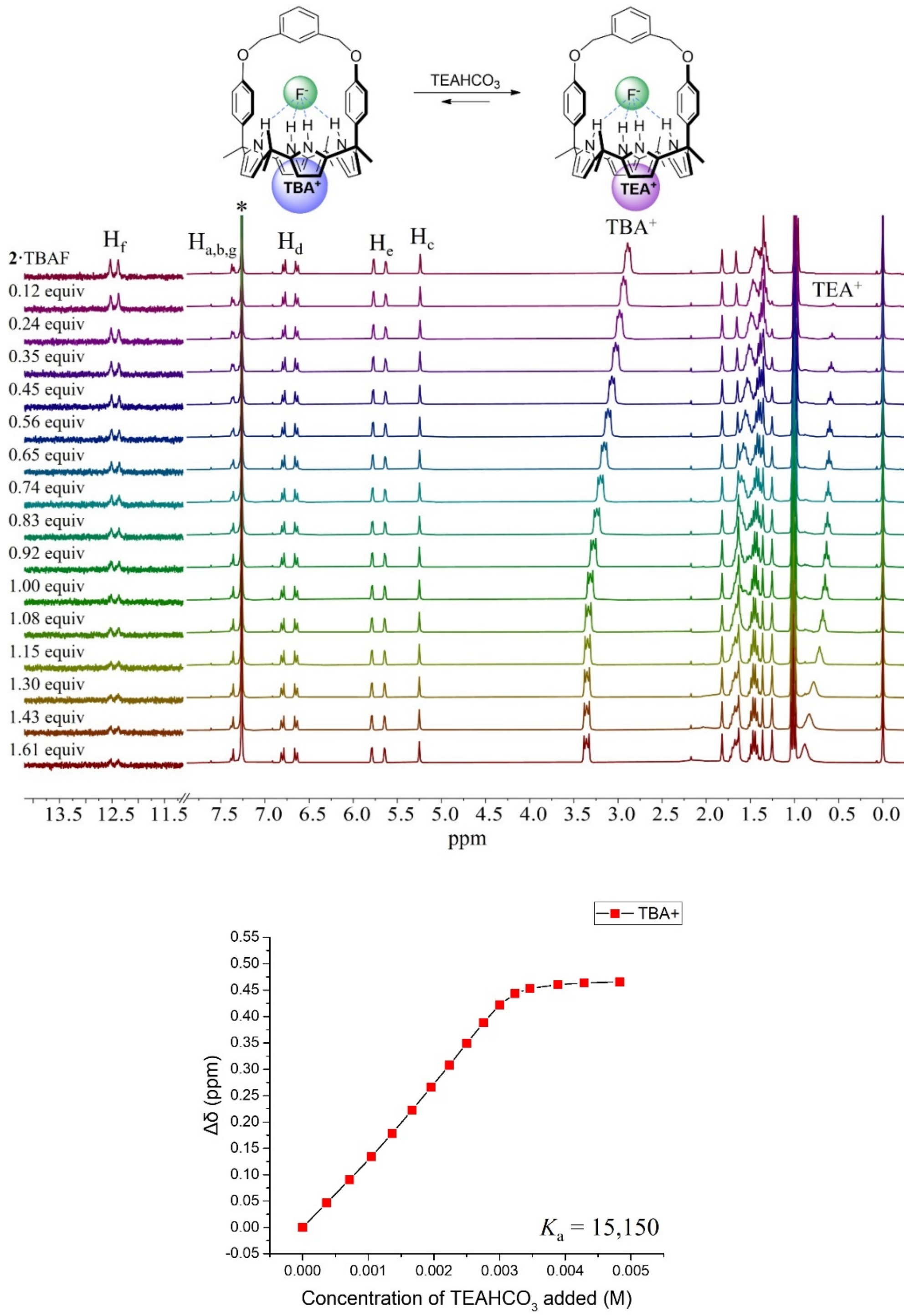

Figure S12. Top: Proposed cation exchange process between TBA^+ an TEA^+. For proton labels see Figure S6. Middle: ^1H NMR spectra recorded during the titration of the TBAF complex of receptor **1** (**1**·TBAF, 3 mM) with $TEAHCO_3$ in $CDCl_3$. Bottom: The corresponding binding isotherm for cation metathesis between TBA^+ and TEA^+. The complex of **1**·TBAF was prepared by adding 3 equiv. of TBAF to the $CDCl_3$ solution of receptor **1**. *Denotes the residual $CHCl_3$ peak from the NMR solvent. The association constant of the TBAF complex of receptor **1** for TEA^+ was obtained using BindFit v5.0 available from URL: "http://app.supramolecular.org/bindfit/".

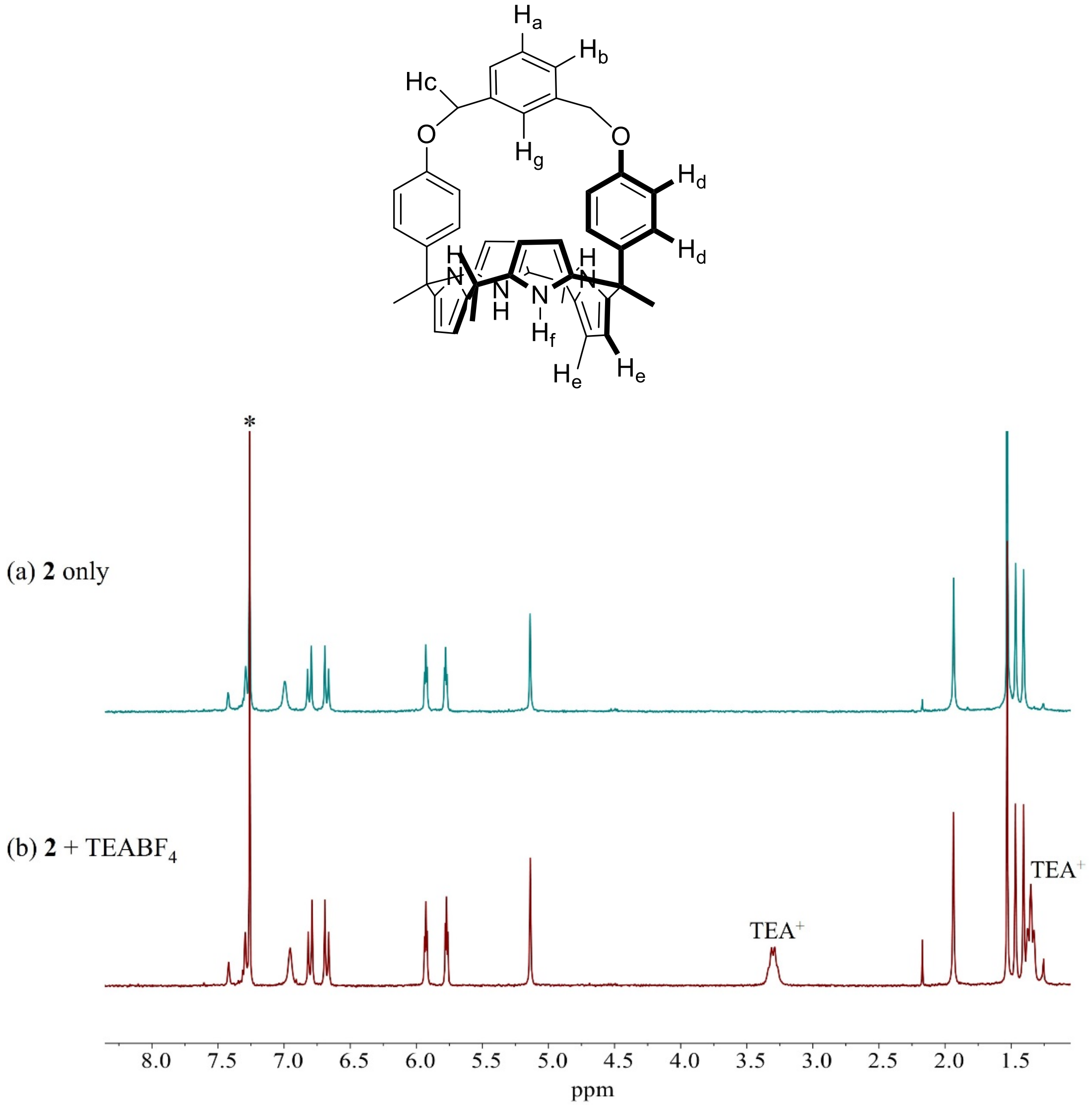

Figure S13. Partial ^{1}H NMR spectra of (a) **2** (3 mM) only and (b) **2** + excess $TEABF_4$ in $CDCl_3$.

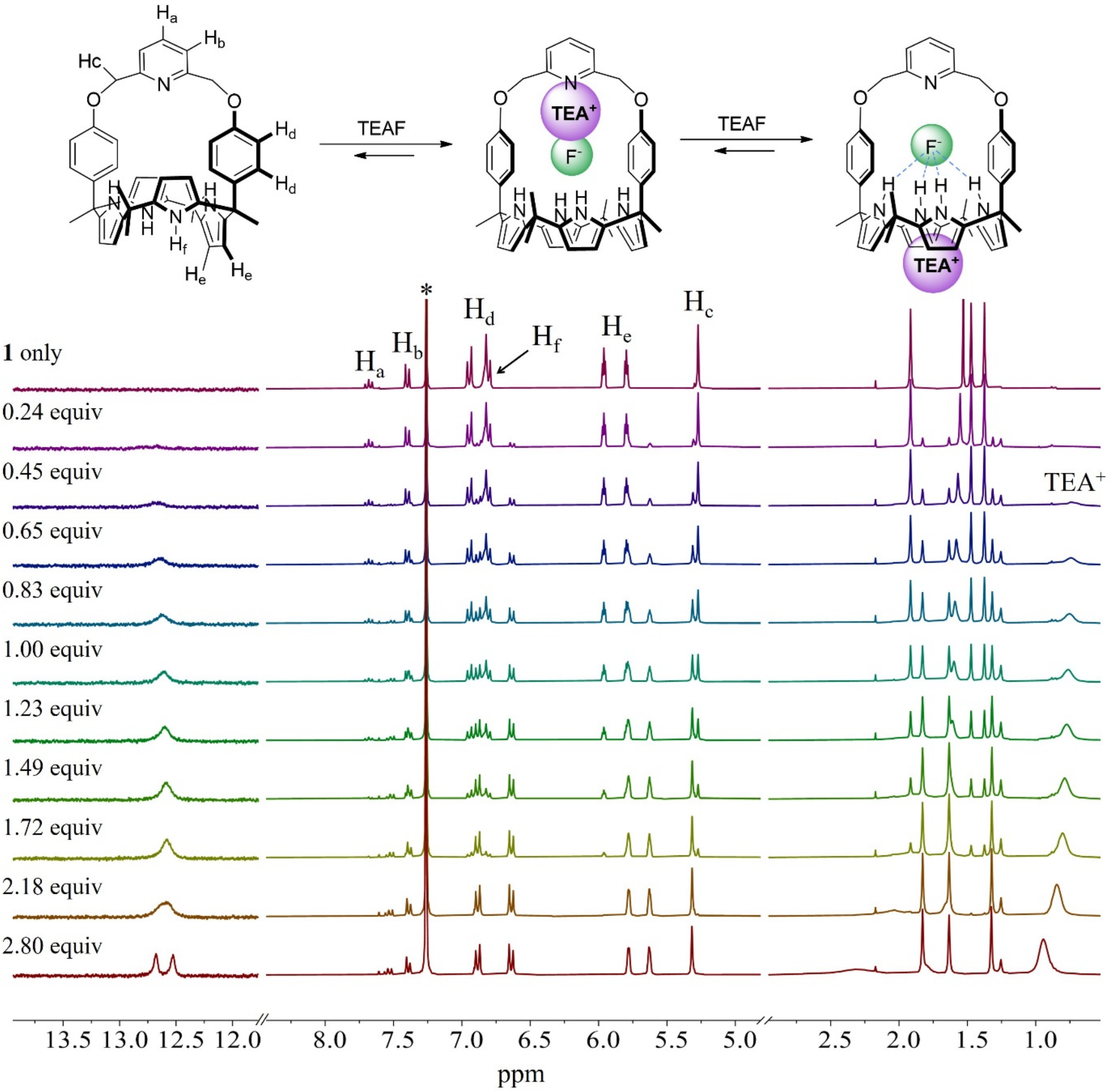

Figure S14. Top: Putative binding modes of receptor **1** with TEAF in $CDCl_3$. Bottom: ^1H NMR spectra recorded during the titration of receptor **1** (3 mM) with TEAF in $CDCl_3$. *Denotes peaks originating from residual $CHCl_3$ present in the NMR solvent.

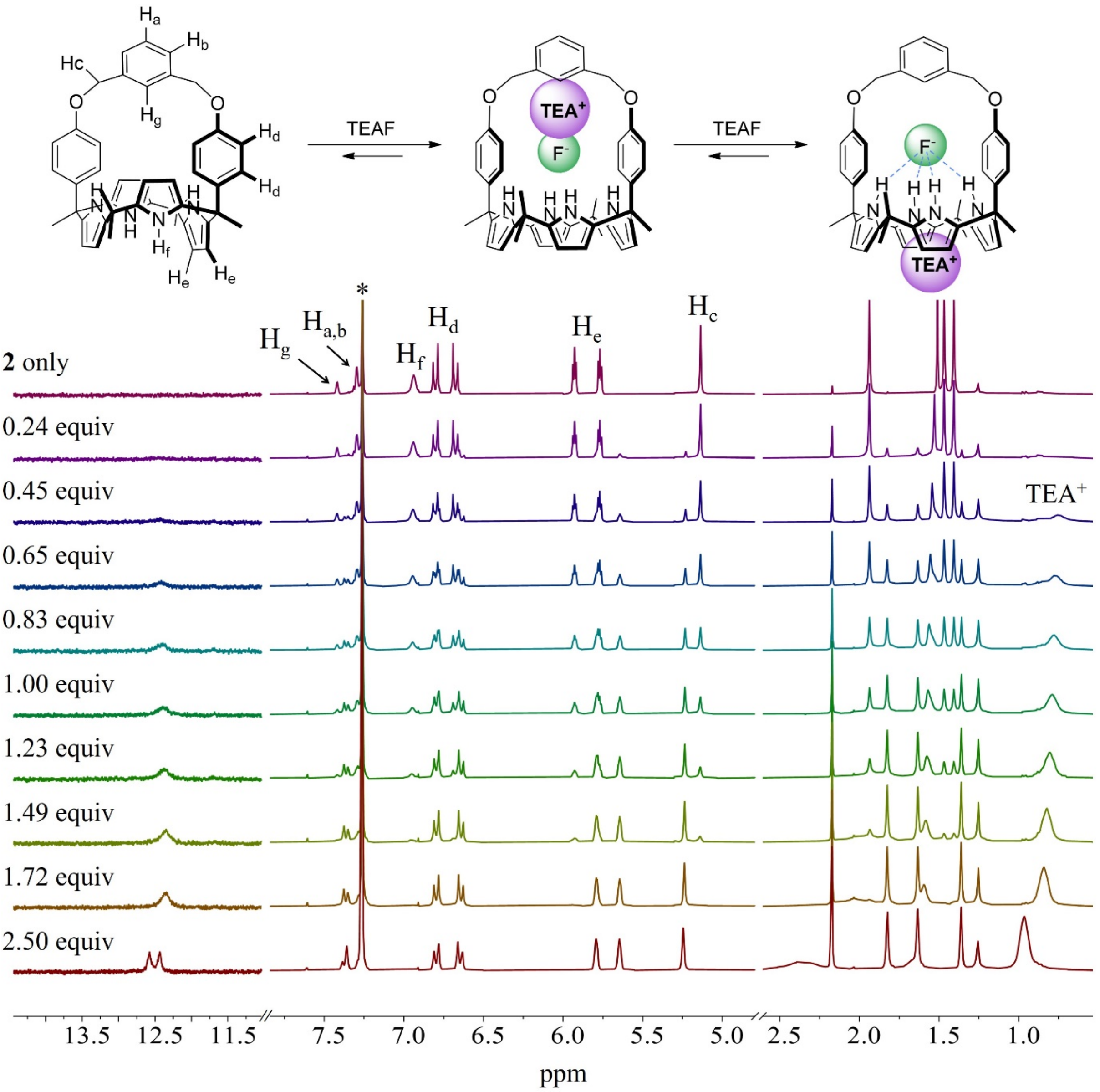

Figure S15. Top: Putative binding modes of receptor **2** with TEAF in $CDCl_3$. Bottom: ^1H NMR spectra recorded during the titration of receptor **2** (3 mM) with TEAF in $CDCl_3$. *Denotes the residual $CHCl_3$ peak from the NMR solvent.

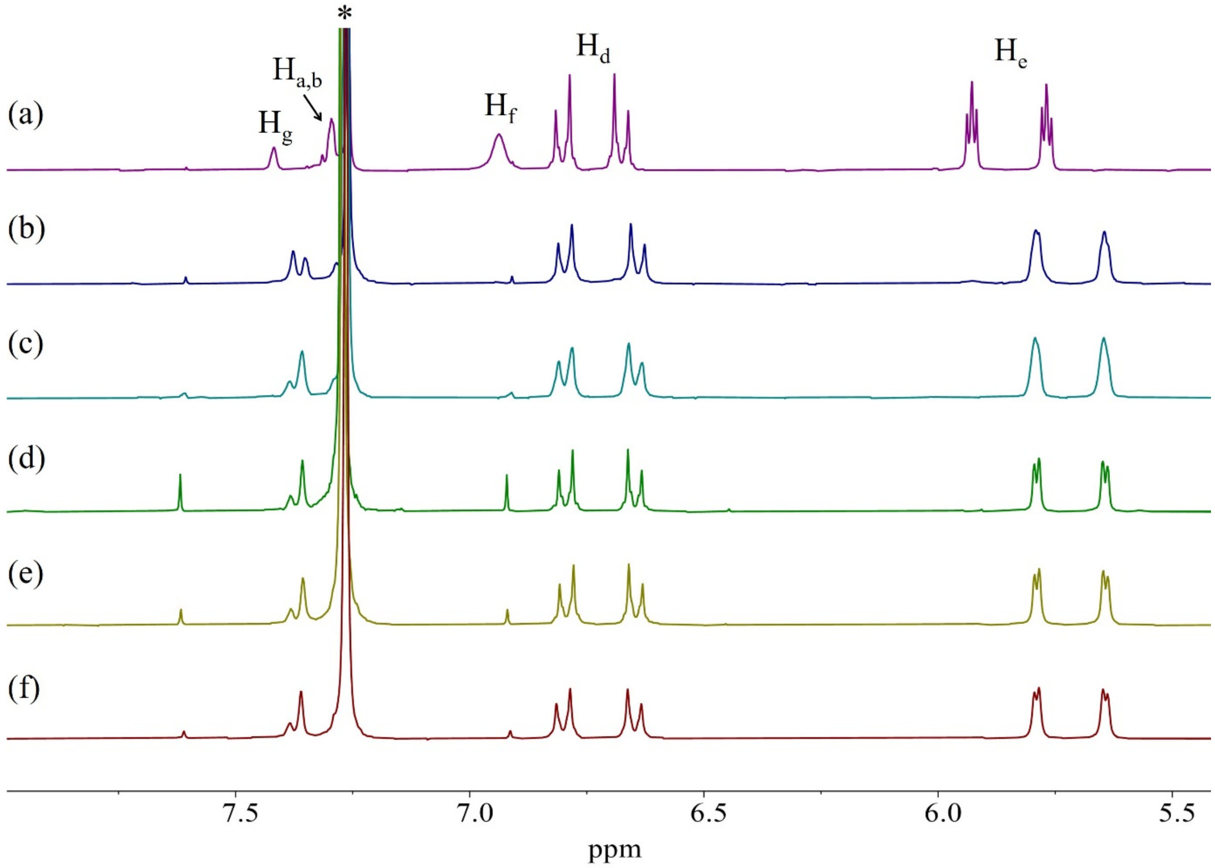

Figure S16. Partial ^{1}H NMR spectra of (a) **2** (3 mM) only, (b) **2** + 1.74 equiv. TEAF, (c) **2** + 2.50 equiv. TEAF, (d) **2**•TEAHCO$_3$ + 1.88 equiv. TBAF, (e) **2**·TEAHCO$_3$ + 3.02 equiv. TEAF, and (f) **2**·TBAF + 1.61 equiv. TEAHCO$_3$. . *Denotes the residual CHCl$_3$ peak from the NMR solvent.

Section S3: Discussion of the interactions between the TEAF ion pair with receptors 1 and 2

During the titrations of receptors **1** and **2** with TEAF, their pyrrolic NH proton signal appeared as a broad singlet with significant downfield shifts before the proton signals corresponding to the ion-free receptors disappear as a result of complete TEAF binding (Figures S14 and S15). This finding was taken as evidence that the hydrogen bonding interactions between the F^- anion (as its TEA^+ salt) and the pyrrolic NH hydrogens of receptors **1** and **2** are relatively weak as compared with what is observed when TBAF is added to the receptors in either their ion-free or $TEAHCO_3$ complexed forms. The further addition of TEAF past the point of initial saturation leads to a splitting of these singlets into doublets. Such a finding is rationalized in terms of strengthened hydrogen bonding interactions between the pyrrolic NH protons of receptors **1** and **2** and the bound F^- anion (Figures S14 and S15). This change is interpreted in terms of conversion of a bound tight ion pair into a receptor separated ion pair complex as shown in Figures S14 and S15 for receptors **1** and **2**, respectively. The initial tight ion pairing was presumed to weaken the hydrogen bonding interactions between the F^- anion and the pyrrolic NH protons of receptors **1** and **2** as evidenced by the lack of NH-F spin coupling. This binding mode is considered kinetically favored. The doublet seen for the N*H* protons upon further addition of TEAF is consistent with strengthened hydrogen bonding interactions between the F^- anion and the pyrrolic N*H* protons. This increase is ascribed to the TEA^+ counter cation being bound to the cone-shaped electron-rich calix[4]pyrrole cavity to form the same receptor-separated ion pair complex produced upon adding F^- to the preformed $TEAHCO_3$ complex (Figures S14 and S15). Evidence for this change in TEA^+ binding mode came from changes in the chemical shift values for the TEA^+ counter cation observed during the titration with TEAF. For instance, the proton signals of the TEA^+ that were seen to resonate at relatively high field prior to saturation, but then underwent noticeable downfield shifts upon the further addition of TEAF (> 2.80 equiv. for **1** and > 2.50 equiv. for **2**). At the same time, the singlet of the N*H* protons (H_f) was split into a doublet (Figures S14 and S15). The chemical shifts corresponding to the aromatic signals were found to be almost identical to those for the TEAF complexes formed by the addition of TBAF or TEAF to the preformed $TEAHCO_3$ complex or by the addition of $TEAHCO_3$ to the TBAF complexes (cf. Figure S16).

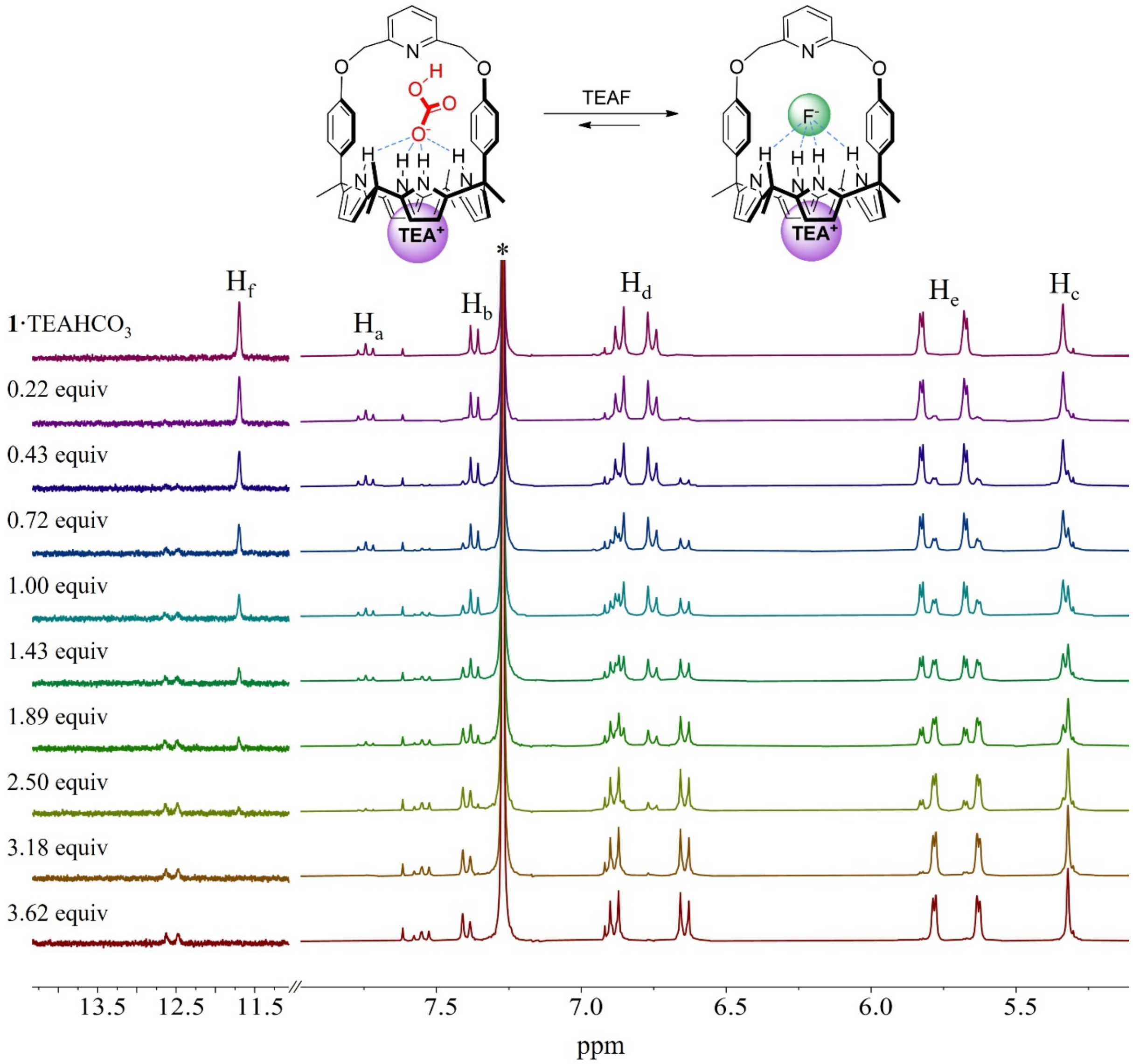

Figure S17. Partial ^{1}H NMR spectra recorded during the titration of the $TEAHCO_3$ complex of receptor **1** (**1**•$TEAHCO_3$, 3 mM) with TEAF in $CDCl_3$. *Denotes peaks originating from residual $CHCl_3$ present in the NMR solvent.

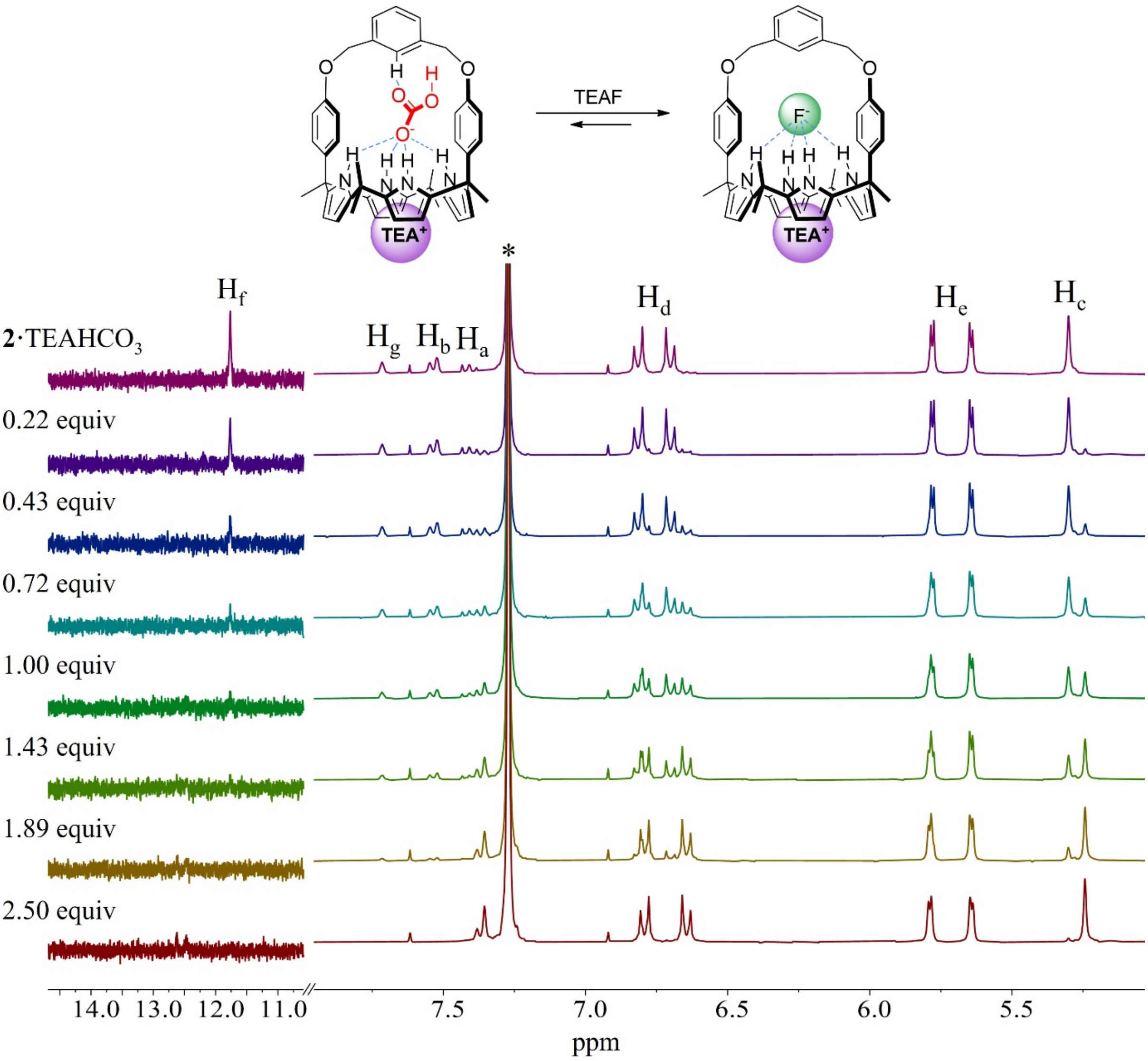

Figure S18. Partial ^{1}H NMR spectra recorded during the titration of the $TEAHCO_3$ complex of receptor **2** (**2**•$TEAHCO_3$, 3 mM) with TEAF in $CDCl_3$. *Denotes the residual $CHCl_3$ peak from the NMR solvent.

X-ray experimental

X-ray experimental for the receptor 1

X-ray experimental for $C_{45}H_{45}N_5O_2$: Crystals grew as large, well formed colorless prisms by slow evaporation from chloroform and methanol. The data crystal was cut from a larger crystal and had approximate dimensions; 0.43 × 0.25 × 0.22 mm. The data were collected on an Agilent Technologies SuperNova Dual Source diffractometer using a μ-focus Cu K_α radiation source (λ = 1.5418 Å) with collimating mirror monochromators. A total of 1021 frames of data were collected using ω-scans with a scan range of 1° and a counting time of 3 seconds per frame for frames collected with a detector offset of -41.6° and 10 seconds per frame with frames collected with a detector offset of 108.3°. The data were collected at 100 K using an Oxford Cryostream low temperature device. Details of crystal data, data collection and structure refinement are listed in Table S1. Data collection, unit cell refinement and data reduction were performed using Rigaku Oxford Diffraction's CrysAlisPro V 1.171.40.53.[2] The structure was solved by direct methods using SHELXT[3] and refined by full-matrix least-squares on F^2 with anisotropic displacement parameters for the non-H atoms using SHELXL-2016/6.[4] Structure analysis was aided by use of the programs PLATON[5], OLEX2[6] and WinGX.[7] The hydrogen atoms were calculated in ideal positions with isotropic displacement parameters set to 1.2 × Ueq of the attached atom (1.5 × Ueq for methyl hydrogen atoms).

The function, $\Sigma w(|F_O|^2 - |F_C|^2)^2$, was minimized, where $w = 1/[(\sigma(F_O))^2 + (0.0785{*}P)^2 + (1.4379{*}P)]$ and $P = (|F_O|^2 + 2|F_C|^2)/3$. $R_W(F^2)$ refined to 0.133, with R(F) equal to 0.0488 and a goodness of fit, S, = 1.01. Definitions used for calculating R(F), $R_W(F^2)$ and the goodness of fit, S, are given below.[8] The data were checked for secondary extinction effects but no correction was necessary. Neutral atom scattering factors and values used to calculate the linear absorption coefficient are from the International Tables for X-ray Crystallography (1992).[9] All figures were generated using SHELXTL/PC.[10] Tables of positional and thermal parameters, bond lengths and angles, torsion angles and figures are found from the Cambridge Crystallographic Centre by referencing CCDC number 2010538.

Table S1. Crystal data and structure refinement for **1**.

Empirical formula	C45 H45 N5 O2	
Formula weight	687.86	
Temperature	100.0(7) K	
Wavelength	1.54184 Å	
Crystal system	monoclinic	
Space group	P 1 21/n 1	
Unit cell dimensions	a = 12.9243(2) Å	α = 90°.
	b = 16.0575(2) Å	β = 103.6360(10)°.
	c = 18.3402(3) Å	γ = 90°.
Volume	3698.89(10) $Å^3$	
Z	4	
Density (calculated)	1.235 Mg/m^3	
Absorption coefficient	0.600 mm^{-1}	
F(000)	1464	
Crystal size	0.43 × 0.25 × 0.22 mm^3	
Theta range for data collection	3.705 to 74.057°.	
Index ranges	-12<=h<=15, -20<=k<=19, -22<=l<=13	
Reflections collected	21929	
Independent reflections	7377 [R(int) = 0.0398]	
Completeness to theta = 67.684°	99.9 %	
Absorption correction	Semi-empirical from equivalents	
Max. and min. transmission	1.00000 and 0.70772	
Refinement method	Full-matrix least-squares on F^2	
Data / restraints / parameters	7377 / 312 / 475	
Goodness-of-fit on F^2	1.031	
Final R indices [I>2sigma(I)]	R1 = 0.0488, wR2 = 0.1294	
R indices (all data)	R1 = 0.0515, wR2 = 0.1328	
Extinction coefficient	n/a	
Largest diff. peak and hole	0.466 and -0.251 e.$Å^{-3}$	

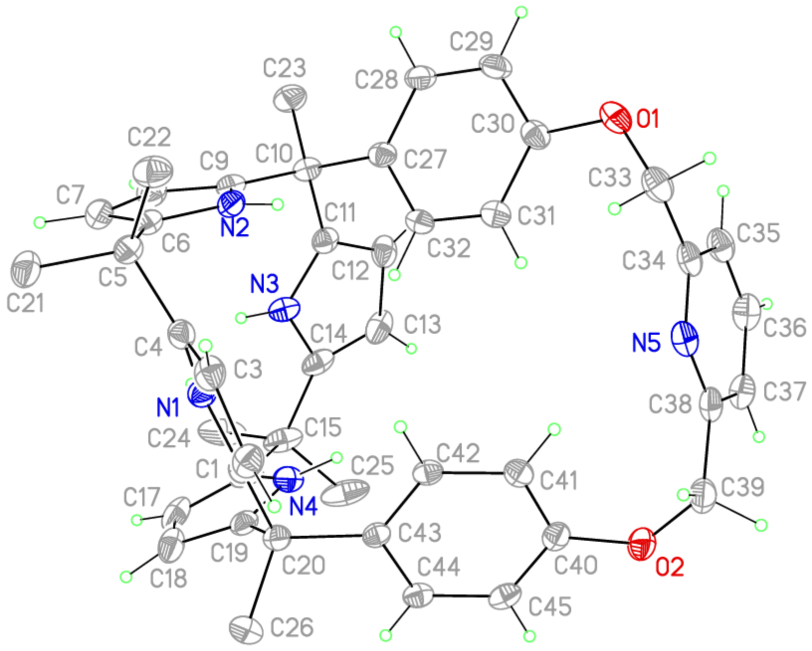

Figure S19. View of **1** showing the atom labeling scheme. Displacement ellipsoids are scaled to the 50% probability level. The methyl group hydrogen atoms have been omitted for clarity.

X-ray Experimental for 1•TEAHCO$_3$.

X-ray Experimental for $C_{45}H_{45}N_5O_2$ – $C_8H_{20}N^{1+}$ HCO_3^{1-} - $CHCl_3$: Crystals grew as colorless prisms by slow evaporation from chloroform and acetonitrile. The data crystal was cut from a larger crystal and had approximate dimensions; 0.37 × 0.19 × 0.068 mm. The data were collected on an Agilent Technologies SuperNova Dual Source diffractometer using a μ-focus Cu Kα radiation source (λ = 1.5418 Å) with collimating mirror monochromators. A total of 308 frames of data were collected using ω-scans with a scan range of 1° and a counting time of 4 seconds per frame using a detector offset of -41.7° and a counting time of 12.5 seconds per frame using a detector offset of 107.1°. The data were collected at 100 K using an Oxford Cryostream low temperature device. Details of crystal data, data collection and structure refinement are listed in Table S2. Data collection, unit cell refinement and data reduction were performed using Agilent Technologies CrysAlisPro V 1.171.40.67a.[2] The structure was solved by direct methods using SHELXT[3] and refined by full-matrix least-squares on F^2 with anisotropic displacement parameters for the non-H atoms using SHELXL-2016/6.[4] Structure analysis was aided by use of the programs PLATON98[5] and WinGX.[7] The hydrogen atoms were calculated in ideal positions with isotropic displacement parameters set to 1.2 × Ueq of the attached atom (1.5 × Ueq for methyl hydrogen atoms.

A molecule of chloroform was disordered and could not be modeled adequately. The scattering due to this molecule was removed by the use of the program, SQUEEZE. The complex resided around a crystallographic two-fold rotation axis. The bicarbonate ion and the 2,6-methoxy-pyridine portion of the structure were disordered about this two-fold axis. The disorder was modeled using features found in OLEX2. The hydrogen atom on the bicarbonate ion could not be located in the difference electron density map and was excluded from the final refinement model.

The function, $\Sigma w(|F_O|^2 - |F_C|^2)^2$, was minimized, where $w = 1/[(\sigma(F_O))^2 + (0.1733*P)^2 + (3.0124*P)]$ and $P = (|F_O|^2 + 2|F_C|^2)/3$. $R_W(F^2)$ refined to 0.256, with R(F) equal to 0.0802 and a goodness of fit, S, = 1.05. Definitions used for calculating R(F), $R_W(F^2)$ and the goodness of fit, S, are given below.[8] The data were checked for secondary extinction effects but no correction was necessary. Neutral atom scattering factors and values used to calculate the linear absorption coefficient are from the International Tables for X-ray Crystallography (1992).[9] All figures were generated using SHELXTL/PC.[10] Tables of positional and thermal parameters, bond lengths and angles, torsion angles and figures are found from the Cambridge Crystallographic Centre by referencing CCDC number 2010539.

Table S2. Crystal data and structure refinement for **1**•$TEAHCO_3$.

Empirical formula	C54 H65 N6 O5	
Formula weight	878.12	
Temperature	100.01(11) K	
Wavelength	1.54184 Å	
Crystal system	tetragonal	
Space group	P 41 21 2	
Unit cell dimensions	a = 15.3125(2) Å	α = 90°.
	b = 15.3125(2) Å	β = 90°.
	c = 23.8680(4) Å	γ = 90°.
Volume	5596.37(19) $Å^3$	
Z	4	
Density (calculated)	1.042 Mg/m^3	
Absorption coefficient	0.533 mm^{-1}	
F(000)	1884	
Crystal size	0.37 × 0.19 × 0.068 mm^3	
Theta range for data collection	3.429 to 73.218°.	
Index ranges	-11<=h<=17, -12<=k<=18, -24<=l<=28	
Reflections collected	10560	
Independent reflections	5480 [R(int) = 0.0240]	
Completeness to theta = 67.684°	99.8 %	
Absorption correction	Semi-empirical from equivalents	
Max. and min. transmission	1.000 and 0.566	
Refinement method	Full-matrix least-squares on F^2	
Data / restraints / parameters	5480 / 122 / 394	
Goodness-of-fit on F^2	1.054	
Final R indices [I>2sigma(I)]	R1 = 0.0802, wR2 = 0.2419	
R indices (all data)	R1 = 0.0891, wR2 = 0.2557	
Absolute structure parameter	-2.4(2)	
Extinction coefficient	0.0014(4)	
Largest diff. peak and hole	0.628 and -0.305 e.$Å^{-3}$	

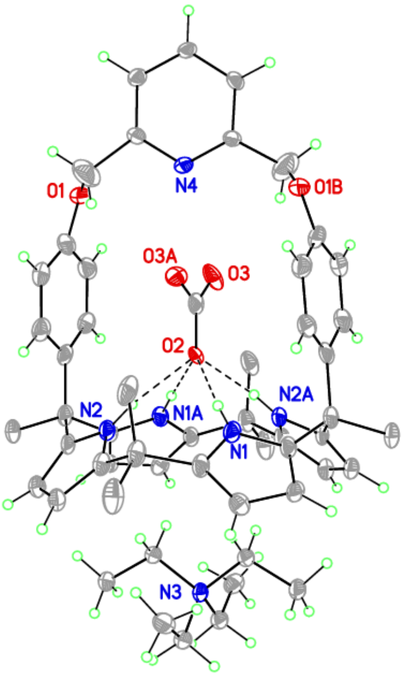

Figure S20. View of the bicarbonate complex **1** showing the heteroatom labeling scheme. Displacement ellipsoids are scaled to the 30% probability level. Some methyl hydrogen atoms have been omitted for clarity.

NMR spectra and HRMS data

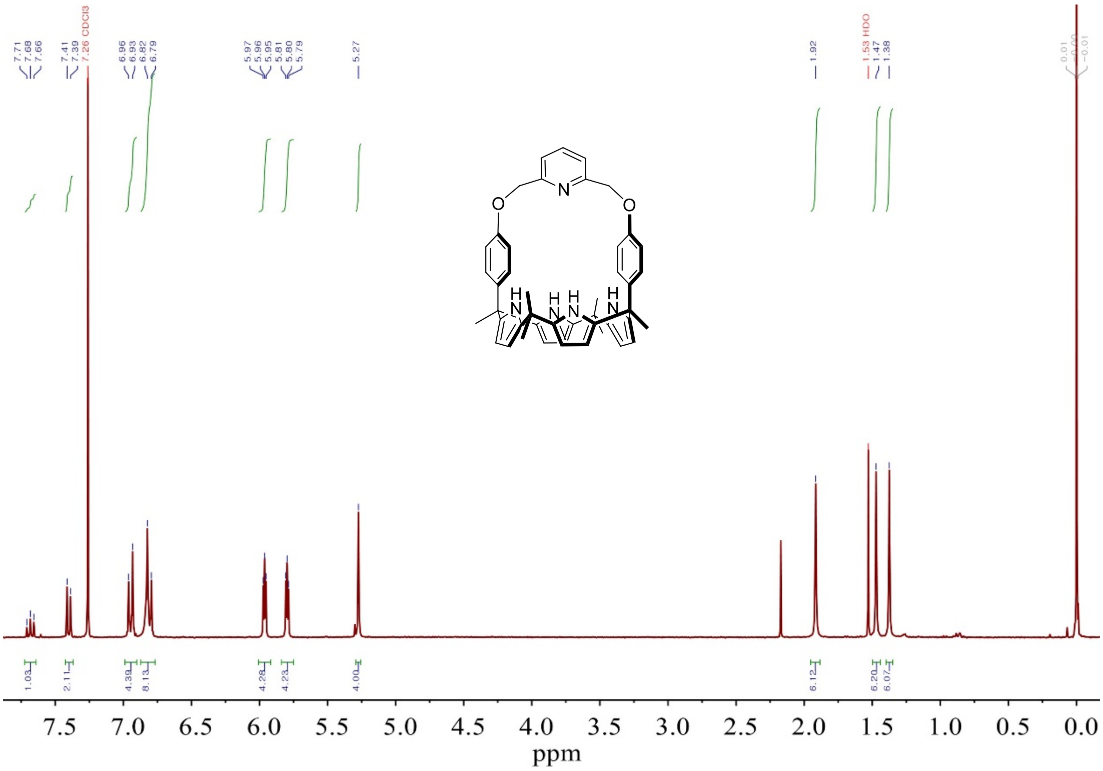

Figure S21. ^{1}H NMR spectrum of **1** recorded in $CDCl_3$.

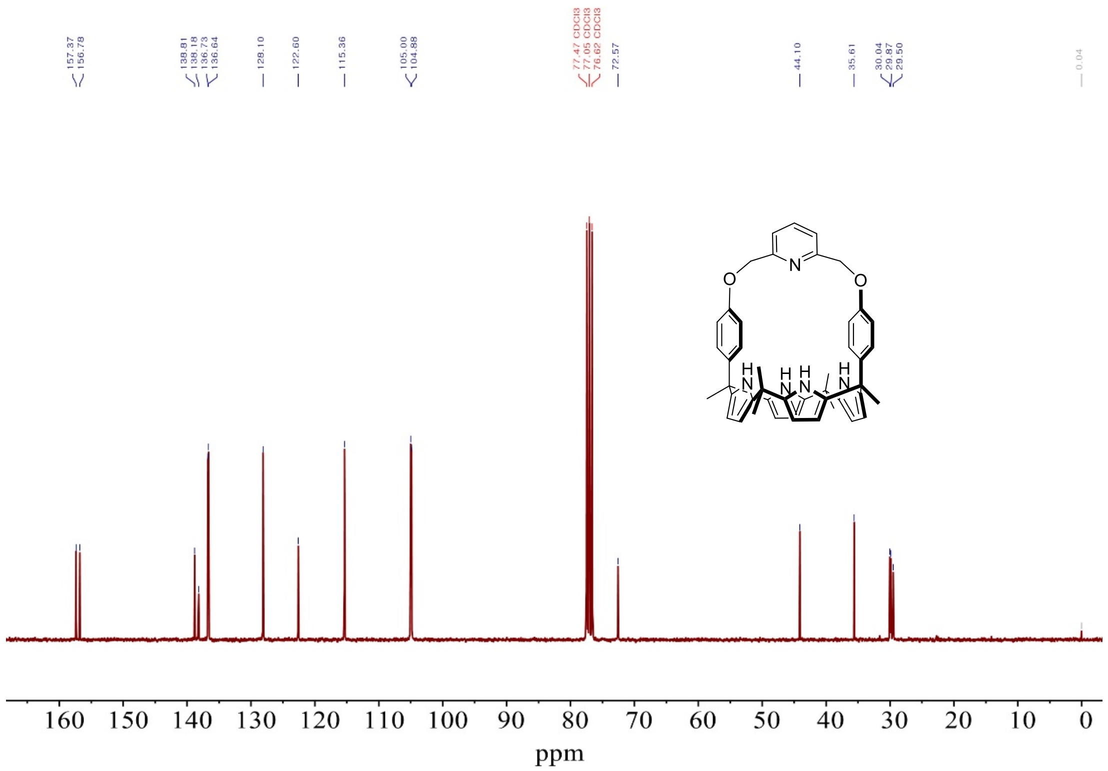

Figure S22. ^{13}C NMR spectrum of **1** recorded in $CDCl_3$.

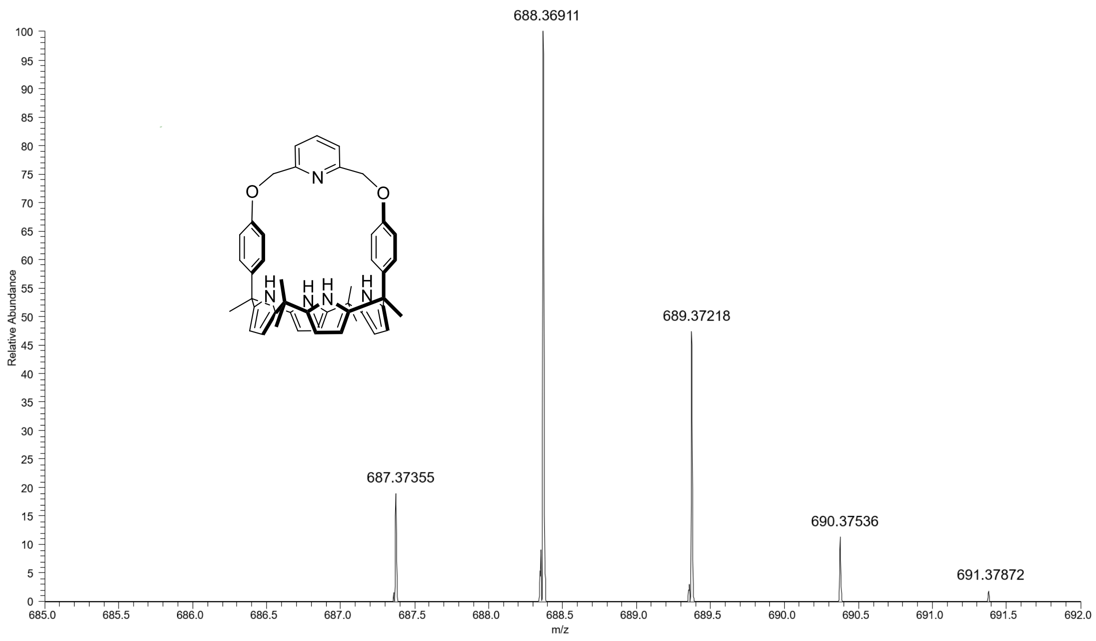

Figure S23. ESI HRMS of receptor **1**.

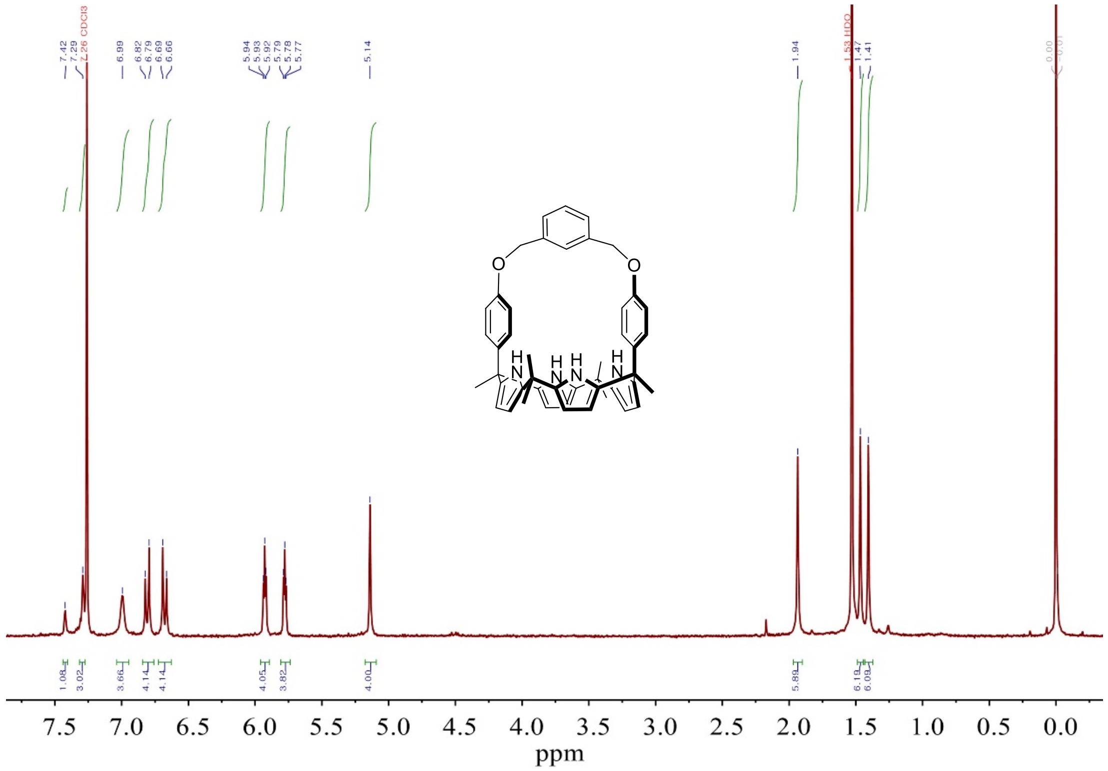

Figure S24. ^{1}H NMR spectrum of **2** recorded in $CDCl_3$.

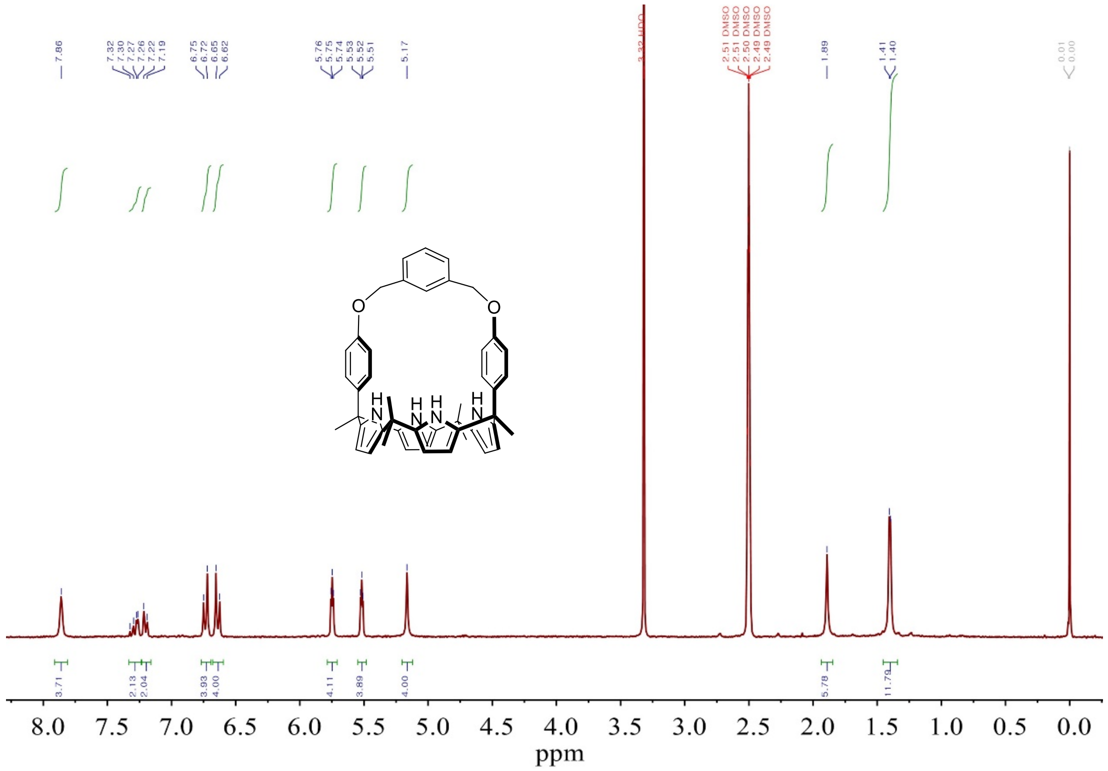

Figure S25. ^{1}H NMR spectrum of **2** recorded in DMSO-d_6.

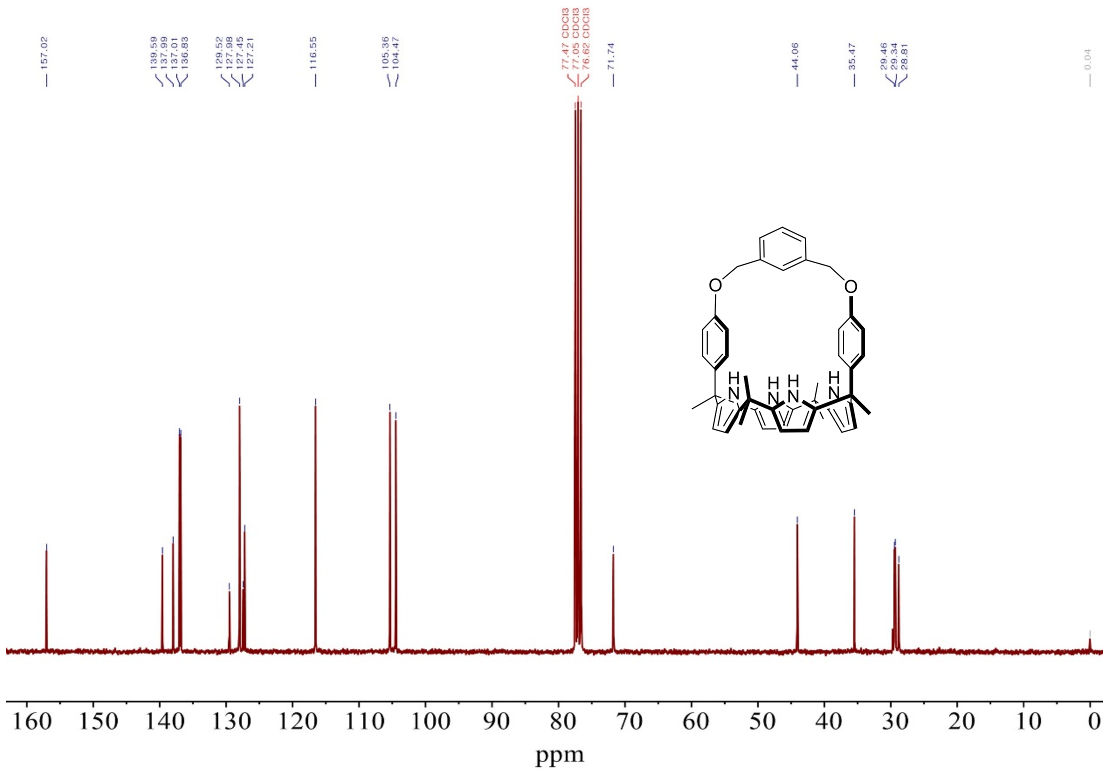

Figure S26. ^{13}C NMR spectrum of **2** recorded in $CDCl_3$.

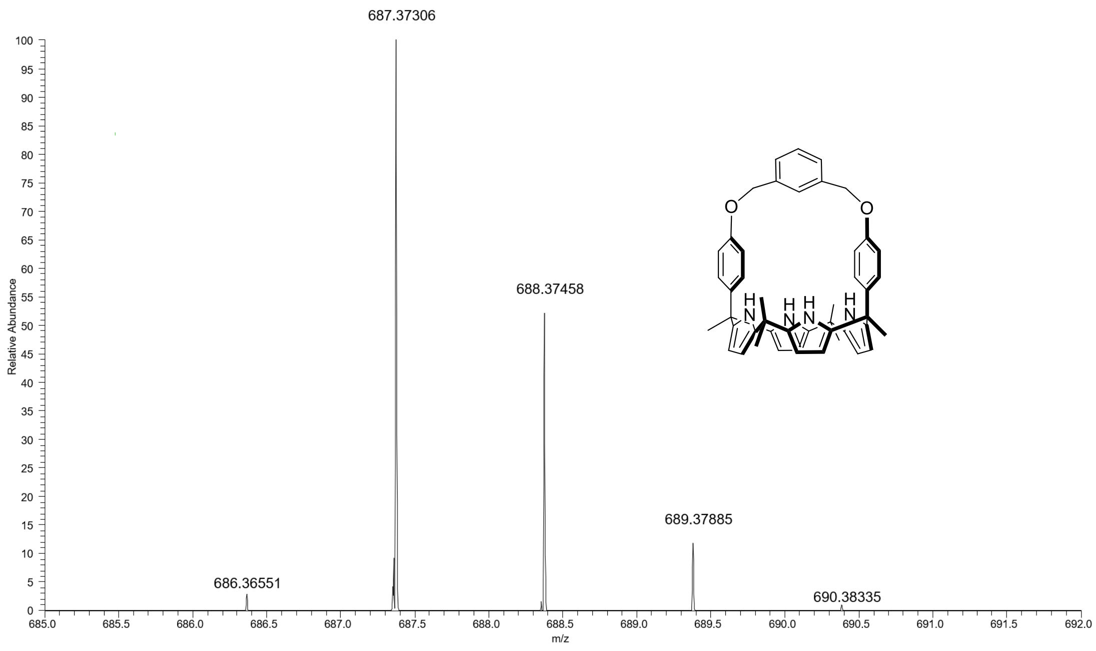

Figure S27. ESI HRMS of receptor **2**.

References

1. J. Yoo, I.-W. Park, T.-Y. Kim and Lee, C.-H. *Bull. Korean Chem. Soc.* 2010, **31**, 630-634.
2. CrysAlisPro. Rigaku Oxford Diffraction (2019). CrysAlicPro Software System, 1.171.40.53.
3. SHELXT. (2015). G. M. Sheldrick, A program for crystal structure solution. Acta Cryst. A71, 3-8.
4. G. M. Sheldrick, (2015). SHELXL-2016/6. Program for the Refinement of Crystal Structures. Acta Cryst., C71, 9-18.
5. A. L. Spek, (2009). PLATON, A Multipurpose Crystallographic Tool. Utrecht University, The Netherlands. Acta Cryst. D65, 148-155.
6. OLEX2. O. V. Dolomanov, L. J. Bourhis, R. J. Gildea, J. A. K. Howard and H. Puschmann, A Complete Structure Solution, Refinement and Analysis Program. J. Appl. Cryst. **42**, 339-341.
7. WinGX 1.64. (1999). An Integrated System of Windows Programs for the Solution, Refinement and Analysis of Single Crystal X-ray Diffraction Data. L. J. Farrugia, J. Appl. Cryst. **32**. 837-838.
8. $R_W(F^2) = \{\Sigma w(|F_O|^2 - |F_C|^2)^2/\Sigma w(|F_O|)^4\}^{1/2}$ where w is the weight given each Reflection. $R(F) = \Sigma(|F_O| - |F_C|)/\Sigma|F_O|\}$ for reflections with $F_O > 4(\Sigma (F_O))$.S $= [\Sigma w(|F_O|^2 - |F_C|^2)^2/(n - p)]^{1/2}$, where n is the number of reflections and p is thenumber of refined parameters.
9. International Tables for X-ray Crystallography (1992). Vol. C, Tables 4.2.6.8 and 6.1.1.4, A. J. C. Wilson, editor, Boston: Kluwer Academic Press.
10. G. M. Sheldrick, (1994). SHELXTL/PC (Version 5.03). Siemens Analytical X-ray Instruments, Inc., Madison, Wisconsin, USA.
